# Hybrid semantic recommender system for chemical compounds in large-scale datasets

**DOI:** 10.1186/s13321-021-00495-2

**Published:** 2021-02-23

**Authors:** Marcia Barros, Andre Moitinho, Francisco M. Couto

**Affiliations:** 1 LASIGE, Departamento de Informática, Faculdade de Ciências, Universidade de Lisboa, 1749–016 Lisboa, Portugal; 2 CENTRA, Departamento de Física, Faculdade de Ciências, Universidade de Lisboa, 1749–016 Lisboa, Portugal

**Keywords:** Recommender system, Chemical compound, Ontology, Semantic similarity

## Abstract

The large, and increasing, number of chemical compounds poses challenges to the exploration of such datasets. In this work, we propose the usage of recommender systems to identify compounds of interest to scientific researchers. Our approach consists of a hybrid recommender model suitable for implicit feedback datasets and focused on retrieving a ranked list according to the relevance of the items. The model integrates collaborative-filtering algorithms for implicit feedback (Alternating Least Squares and Bayesian Personalized Ranking) and a new content-based algorithm, using the semantic similarity between the chemical compounds in the ChEBI ontology. The algorithms were assessed on an implicit dataset of chemical compounds, CheRM-20, with more than 16.000 items (chemical compounds). The hybrid model was able to improve the results of the collaborative-filtering algorithms, by more than ten percentage points in most of the assessed evaluation metrics.

## Introduction

Chemical entities/compounds, defined as “physical entities of interest in chemistry including molecular entities, parts thereof, and chemical substance” [1], are growing in number and complexity, generating large datasets, challenging for the researchers to explore deeply. Recommender systems (RS) may be a feasible solution for this challenge by identifying new entities to explore, for example, by suggesting entities not yet studied by the researchers based on their past investigation projects. However, the recommendation of chemical compounds of interest has not been widely explored [2, 3]. One challenge to include RS in compound databases is the lack of available datasets with the preferences of the researchers about the chemical compounds for assessing the RS. For example, it is not easy to explicitly know if a specific researcher had interest in the study of a chemical or not. More recently, alternatives have emerged with the development of datasets consisting of data collected from implicit feedback [4, 5]. These datasets do not contain the explicit interests of the users, as other famous datasets, such as Movielens [6]. Instead, this information is extracted from their activities, mostly from the scientific literature, which remains the main method for disseminating scientific work.

Datasets of explicit or implicit feedback require different recommender algorithms, especially because implicit feedback has significant downgrades, such as the lack of negative feedback and unbalanced ratio of positive vs. unobserved ratings [7, 8]. When dealing with implicit feedback datasets, the solution involves applying learning to rank (LtR) approaches. LtR consists in, given a set of items, identify in which order they should be recommended [9].

In RS, the main approaches are Collaborative-Filtering (CF) and Content-Based (CB) [10]. CF uses the similarity between the ratings of the users, and CB uses the similarity between the features of the items. CF is divided into two methods, memory-based and model-based [11]. Memory-based methods deal with the recommendation problem by finding the most similar users based on the ratings of the items. If two users tend to rate the same items in the same way, they will probably like the items seen by each other. Model-based methods use machine learning and data mining for predicting the ratings or for assigning a score to each item by filling the rating matrix blank spaces (unknown ratings). One of the most used methods is matrix factorization since it leverages all row and column correlations in one shot to estimate the entire data matrix [12]. With model-based methods, it is more difficult to explain the recommendations.

CF approaches cannot deal with new items or new users in the system, i.e., items and users without ratings (cold start problem). CB does not suffer from the cold start problem for new items since this approach only needs the features that characterize them to compare with the features of the items that the user already saw or liked. Thus, even if the new item does not have a single rating in the entire dataset, it may still be recommended. However, CB needs a list of features for the items, which varies from field to field. To deal with CF and CB challenges, we can develop hybrid RS, which are the assembling of CF and CB. One of the most common forms of creating hybrids is by a weighted technique, where the scores of the different algorithms are combined into a unique final score [13].

One of the challenges of CB approaches is related to which features to use for finding similar items. Some items have obvious features. For example, when our items are movies, the features used to find similar items may be the genre, director, and authors. In other fields, the task of finding features for the items is not that obvious. Thus, one of the tools used by CB for this purpose are ontologies [14], which provide controlled vocabularies of terms and definitions to represent the entities of a specific field of study [15, 16].

The notion of ontology is not new and has long been used for classifying and describing concepts. At the time of the rising of the semantic web, ontologies were adapted to computational reasoning and knowledge sharing since their structured format (triplets of subject, predicate and object) makes them ideal for computer processing. More recently, ontologies were adapted to the biological/biomedical domain. Some examples of well-known bio-ontologies are the Chemical Entities of Biological Interest (ChEBI) [17, 18], the Gene Ontology (GO) [19, 20], and the Disease Ontology (DO) [21, 22]. Bio-ontologies are particularly important for providing a unique identifier for biomedical entities. The name of biomedical entities may change over time, and different researchers may refer to them differently. One of the advantages of the ontologies is storing lists of these descriptors. Considering, for example, the chemical entity caffeine [23]. This entity is identified in the ontology with the primary name **caffeine**, primary ID **CHEBI:27732** and it has an extended list of synonyms:

- 1,3,7-Trimethyl-2,6-dioxopurine
- 1,3,7-Trimethylpurine-2,6-dione
- 1,3,7-Trimethylxanthine
- 1,3,7-Trimethylxanthine
- 1-Methyltheobromine
- 3,7-Dihydro-1,3,7-trimethyl-1*H*-purin-2,6-dion
- 7-Methyltheophylline
- Anhydrous caffeine
- Cafeín
- Caféine
- CAFFEINE
- Caffeine
- Caffeine
- Coffein
- Guaranine
- Koffein
- Mateína
- Methyltheobromine
- Teína
- Thein
- Theine

**Fig. 1:**
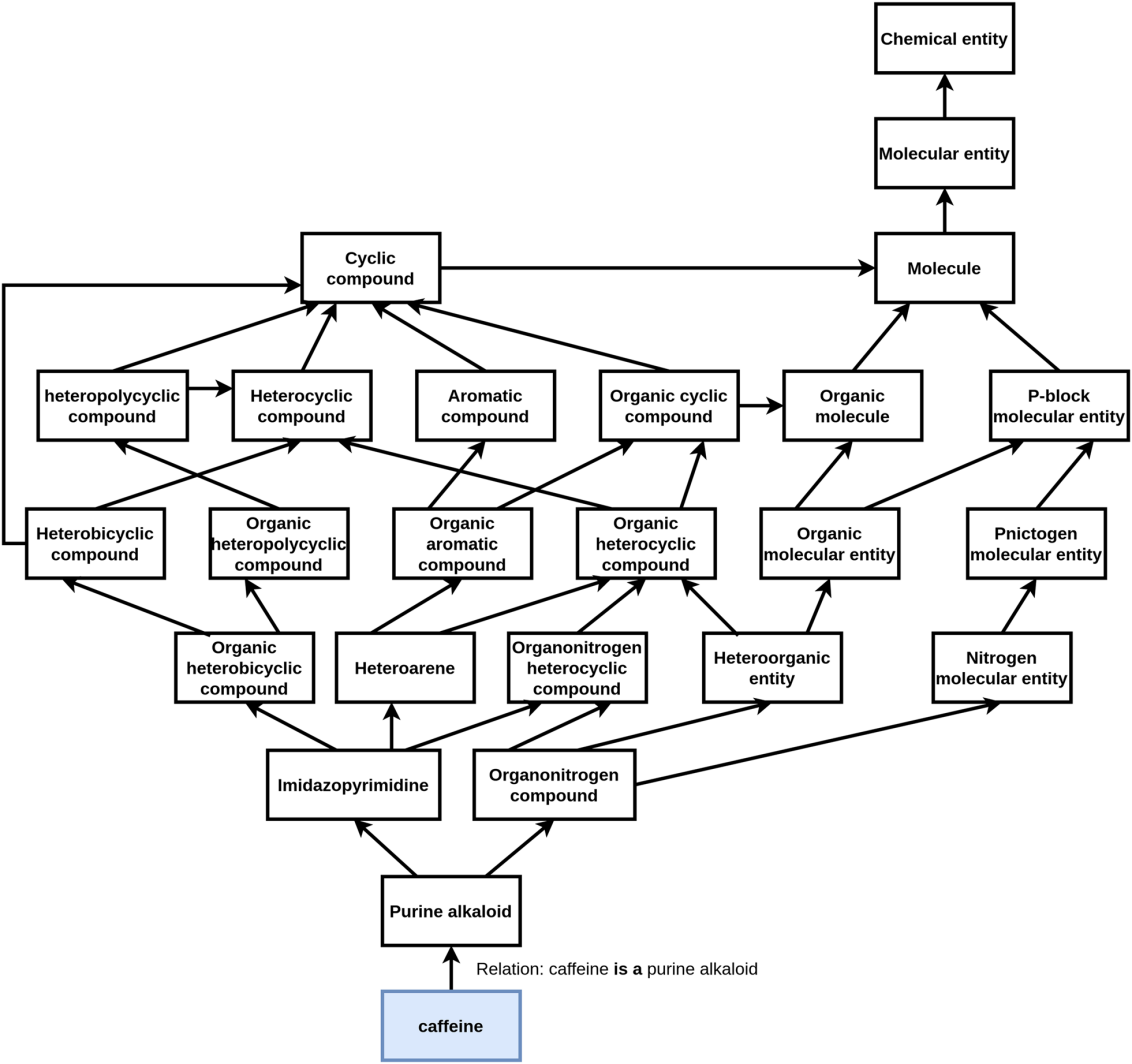
Knowledge graph for caffeine. Knowledge graph for the entity caffeine, adapted from ChEBI

Thus, when a researcher is interested in scientific articles about Koffein, we can use the ontology for identifying all its synonyms and retrieve all the articles that mention them instead of just limiting the search to the given descriptor. Another significant advantage of the ontologies is that we can relate the entities through their semantic similarity, a measure based on the ontology’s semantic structure. Figure 1 shows the knowledge graph adapted from ChEBI for the chemical compound *caffeine*. As we can see in the graph, the relations are defined based on the semantics of the entities, for example, *caffeine* is a *purine alkaloid*. We can use these relations to calculate how much two entities are semantically similar, for example, considering their common ancestors.

Several works have used the semantic similarity between the entities of an ontology. In Ferreira and Couto [24], the authors developed a hybrid method for classifying chemical compounds based on structural and semantic similarity. This work concluded that using semantic similarity improves the classification of the chemical compounds and the best results were obtained when the weight of semantic similarity was higher than two thirds (71%) and the weight of the structural similarity less than one third (29%). More recently, Wang et al. [25] used the structural similarity and the ChEBI semantic similarity assembled into a hybrid for predicting compounds subtracts suitable for membrane transporters. Other studies used the semantic similarity of ChEBI entities for recognition and confirmation of chemical compounds found in research documents [26, 27]. In our work, we propose using the ontologies as a source of features that characterize the scientific items to find similar items for recommendation.

The field of RS is broad, and its approaches are applied to several domains, such as movies [28], books [29], and e-commerce [30]. In the Chemistry domain, RS have been generally used in studies related to drugs, for example, for new drugs design [31], and for finding candidate drugs for diseases [32]. Boström et al. [31] used RS for recommending reagents for new drugs, based on the experience of other chemists. The dataset used in this study, despite interesting, is not available. Hao et al. [32] applied RS techniques for recommending targets to drugs. The datasets used has the format of target-drug pairs, but it does not contain any information about the researcher choices. Most recently, Sosnina et al. [33] used RS approaches to discover new antiviral drugs, extracting compounds from ChEMBL [34], a database of molecules with drug-like properties. The dataset used has the format of compound-viral species-interaction value. The authors explain how the dataset was created, but they do not provide the dataset. Other RS applications in Chemistry may be found in Ishihara et al. [2], which describes the use of CF methods for creating possibilities for new chemical compounds. The dataset is not available. Seko et al. [3] uses RS techniques also for the discovery of new inorganic compounds. The authors used the features of chemical relevant compositions to predict if a certain composition is a good candidate to inorganic compound. If the system predicts a composition as being a new compound, it recommends this composition to further studies. The authors provide some additional material, but not the final dataset used in the RS. Once again, this study does not use a dataset of user, item, rating, and it does not have any information about the preferences of the researchers.

None of the previous studies reported the use of ontologies, as opposed to the studies presented below, in which the use of ontologies enhanced the CF approaches. Liao et al. [35] created a RS for recommending English collections of books in a library. The authors developed PORE, a personal ontology recommender system, which consists of a personal ontology for each user and then applying a CF method. Sieg et al. [36] also used an ontology for creating users’ profiles for the domain of books. They calculated the similarity, not between the ratings of the users, but based on the interest scores derived from the ontology. Shambour and Lu [37] developed a Trust–Semantic Fusion approach, tested on movies and Yahoo! datasets. Their approach incorporates semantic knowledge to the items’ primary information, using knowledge from the ontologies.

Ostuni et al. [38] presented a solution for the top@k recommendations (list of size k with the most relevant items for a user, predicted by the recommendation algorithm) specifically for implicit feedback data. The authors developed the Spank—semantic path-based ranking. They extracted path-based features of the items from DBpedia and used LtR algorithms to get the rank of the most relevant items. They tested the method on music and movies domains. Al-Hassan et al. [39] developed a new semantic similarity measure, the Inferential Ontology-based Semantic Similarity. The new measure improved the results of a user-based CF approach, based on tests on the tourism domain. Most recently, Nilashi et al. [40] developed a Hybrid RS tested on the movies domain. The method used Single Value Decomposition for dimensionality reduction for the item and user-based CF, and ontologies for item-based semantic similarity, improving the CF results. They do not deal with implicit data.

For datasets of implicit feedback, there are two CF algorithms which have been particularly popular, Alternating Least Squares (ALS) [41] and Bayesian Personalized Ranking (BPR) [7]. ALS is a latent factor algorithm that addresses the confidence of a user-item pair rating, which goal is to minimize the least squares error of the observed ratings by factorizing the ratings matrix in user and item matrix. ALS has the advantage of being easily parallelized. Some recent studies focused on speeding up the implementation of this algorithm [42, 43]. Another study developed a recommender system for movies based on ALS using Apache Spark [44]. BPR is also a latent factor algorithm, but it is more appropriate for ranking a list of items. BPR does not just consider the unobserved user-item pairs as zeros but also discerns the preference of a user between an observed and an unobserved rating. Several studies have been using BPR in the recommendation of items from implicit feedback datasets. Bi et al. [45] presented a deep neural network model based on Stack Denoising Auto-Encoder and BPR. Zhao et al. [46] proposed a social distance-aware BPR model for social network recommendations. Zhang et al. [47] presented a solution for the recommendation of restaurants, based on deep learning and BPR, for multi-source datasets of implicit feedback.

Here we present a new hybrid semantic recommender model for recommending chemical compounds that uses semantic similarity and deals with implicit feedback data, of which a prototype has been presented in [48]. The system here presented is now capable of dealing with thousands of items, and the results represent an improvement over top@k in several evaluation metrics. The hybrid model has two modules, one CF and one CB. The CF module addresses the implicit feedback datasets by applying ALS or BPR, and the CB module explores the semantic similarity of the chemical compounds. The Hybrid model combines the outcomes of the CF and CB modules.

The main contributions of this work are:

- A recommender framework for recommending chemical compounds;
- A new CB semantic recommender algorithm named ONTO based on ontologies;
- A new Hybrid recommender algorithm for datasets of implicit feedback;
- A dataset with the semantic similarity between more than 16.000 chemical compounds;
- A faster semantic similarity calculation for DiShIn library.

The framework developed for this work, as well as all the data, is available at https://github.com/lasigeBioTM/ChemRecSys.

## Methods

### Workflow of the proposed model

**Fig. 2:**
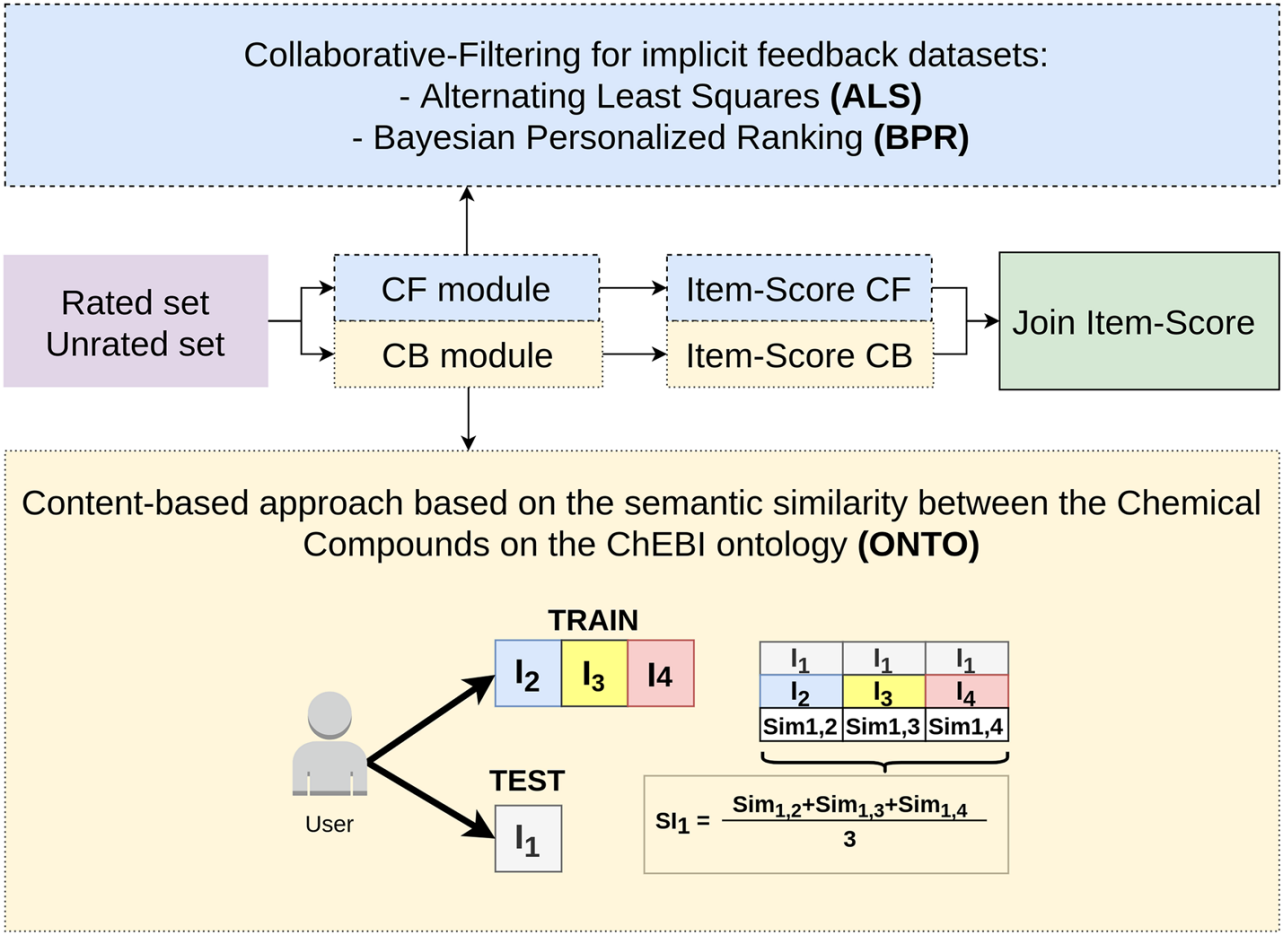
Hybrid model. Workflow of the Hybrid semantic recommender model

In this work we propose a Hybrid recommender model, featuring two modules: CF and CB. Figure 2 shows the general workflow of the model.

The input data used in this model, better described in “Experiments” section, has the format of <user,item,rating>. The unrated set represents the items we want to rank to provide the best recommendations in the first positions to a user. The rated set are the items the users already rated. Since we will split the data into train and test, lets call training set to the rated set and testing set to the unrated set. Both training and testing sets are the input for the CF and CB modules. Using CF algorithms for implicit feedback datasets, the CF module gives a score for each item in the test set. The CB module uses semantic similarity for providing a score for the items in the test set. In the last step, the scores from CF and CB modules are combined and sorted in descending order.

For the CF module, we selected two CF recommender algorithms for recommending data collected from implicit feedback, Alternating Least Squares (ALS) [41] and Bayesian Personalized Ranking (BPR) [7], both implemented in the library *Fast python collaborative filtering for implicit datasets* (implicit) [49]. These algorithms and the implementation in the implicit library are suitable for the type of dataset we are using and they were already used with similar datasets, i.e., recommendation datasets of implicit feedback, especially for recommending music playlists [50, 51]. ALS and BPR are used separately in the CF module. The goal is to verify which combination of CF(ALS or BPR)/CB achieves the best recommendations results. The CF module outputs a score, $\text {S}_{\text{CF}}$, for each test item.

To the CB module, we developed a new algorithm, called ONTO, which is based on the semantic similarity between the items in the ChEBI ontology. This module assigns a score $\text {S}_{\text{CB}}$ to each item in the test set, calculating the semantic similarity between each item in the train and the test sets, as shown in Fig. 2. The semantic similarity allows measuring how close two entities are in a semantic base. When using ontologies, the semantic similarity may be measured, for example, by calculating the shortest path connecting the nodes of two entities. For calculating the similarity, we used DiShIn [52, 53], a tool for calculating semantic similarities between the entities represented by an ontology. DiShIn provides three similarity measures: Resnik [54], Lin [55], and Jiang and Conrath (JC) [56]. All the previous measures are based on the information content of the entities, given by the probability of the entity appears in the ontology, and in the shared information content, calculated from the common ancestors. Resnik and Lin are real similarity measures, whereas JC is a distance measure, posteriorly converted to similarity. Lin and JC have a range between zero and one. The higher the value, the more similar the entities are. The ONTO algorithm is described in Algorithm 1.
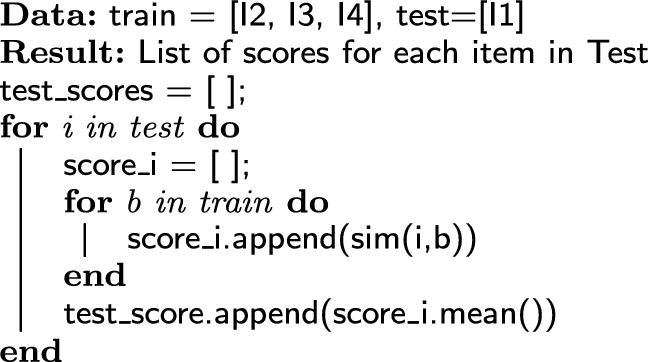


ONTO receives as input two lists of items, train and test. The train data are the items we know the user already saw. The test data contains the items we want to know if suitable for recommending to a user. Thus, for each item in the test set, the ONTO algorithm finds the similarity to each item in the train set and calculates the mean of the similarities, as expressed by Eq. 1.

1 $$S_{\text{CBI}1} = \frac{Sim_{1,2} + Sim_{1,3} + \cdots + Sim_{1,n}}{m}$$

In Eq. 1, $S_{\text{CBI}1}$ is the score for item 1, which is a test item, calculated through the ONTO algorithm, and $\text {Sim}_{1,2}$, $\text {Sim}_{1,3}$, $\text {Sim}_{1,\text{n}}$ are the semantic similarities between item 1 and items 2, 3, …, n, respectively. 2, 3 and n are train items, and m is the number of train items.

Whereas the CF module uses all the ratings from the train set to train the model, CB module only takes into account the ratings of each user. ONTO algorithm does not use any real rating of the test items when calculating the score for each item in the test set, thus we do not have the problem of introducing bias in the results.

The final score for each item in the test set in the Hybrid model is the ensemble of the scores obtained from the CF algorithms, ALS or BPR, and the score obtained by the ONTO algorithm [13]. We used a weighted method, weighting the components heuristically according to two different metrics. Metric1 is represented in Eq. 2 and it multiplies the scores from CF and CB approaches. Metric2 is represented in Eq. 3 and it calculates the mean of the scores.

2 $$Metric1= S_{\text{CFI}1}\times S_{\text{CBI}1}$$

3 $$Metric2= \frac{ S_{\text{CFI}1} + S_{\text{CBI}1}}{2}$$

$\text {S}_{\text{CFI}1}$ is the score obtained for item 1, depending on the CF algorithm that we are using (ALS or BPR for our case study), and $\text {S}_{\text{CBI}1}$ is the score for item 1 obtained with the CB algorithm. Metric2 (Eq. 3) is a more standard approach, however, Metric1 (Eq. 2) allows that items that are really outstanding in one of the algorithms are recommended. Our goal is to prove that by combining both modules, we can improve the results of each module separately.

### Evaluation

There are several methods for evaluating the performance of a RS, depending on the available resources and on the goal of the RS itself. If we have the RS running on a platform, such as YouTube [57] or IMDB [58], we may perform online tests by implementing two algorithms, randomly attributing them to the users, and measuring the recommendations’ clicking rate. However, in most cases, we have only access to offline datasets, i.e., datasets with the past information of the users’ preferences. Despite the disadvantage of not having access to the users’ immediate preferences, using offline datasets give us the chance to test and evaluate new recommendation algorithms without the extra work of developing an online platform and interacting with real users. Also, testing the algorithms offline gives us an indication of the best algorithm to be posteriorly implemented in online platforms. Thus, offline evaluation requires a dataset with the users’ preferences for splitting into train and test sets. The goal is to predict the best items for each user and then use the test set for confirming if the recommended items are relevant for the user [59, 60].

Depending on the goal of the algorithm, the type of evaluation will be different. There are algorithms whose goal is to predict the rating a user would give to an item, and other whose goal is to recommend a ranked list of items, i.e., the top@k items, where k is the size of the list. In the first case, these algorithms are evaluated for the predicted rating, using metrics such as Root Mean Squared Error (RMSE). RMSE measures the differences between the real rating of an item, and the rating predicted by a recommender algorithm, for all *n* items being analyzed.

In the second case, when the algorithms return a ranked list of items, these may be evaluated for the number of relevant items recommended, for example, through Precision (Eq. 4), Recall (Eq. 5), and F-Measure (Eq. 6), and for the quality of the ranking, through Mean Reciprocal Rank (Eq. 7) and Normalized Discounted Cumulative Gain (Eq. 9).

4 $$Precision@k= \displaystyle \frac{relevant\_items@k}{k}$$

5 $$Recall@k= \displaystyle \frac{relevant\_items@k}{total\_relevant\_items}$$

6 $$F\_measure@k= \displaystyle 2\times \frac{Precision\times Recall}{Precision+Recall}$$

7 $$MRR= \frac{1}{n\_users} \sum _{i=1}^{n\_users} \frac{1}{rank_i}$$

8 $$DCG= \sum _{i=1}^{n}\frac{relevance_i}{\log _{2}(i+1)}$$

9 $$nDCG= \frac{DCG}{iDCG}$$

Precision@k provides a measure of the relevant items recommended in the top@k list, recall@k the number of relevant items recommended in the top@k list, and f-measure provides an harmonic mean of precision and recall. The MRR evaluates in which position the first relevant item appears. The nDCG is an evaluation method which compares the ideal ranking of a test set (iDCG), with the ranking assigned by the recommendation algorithm (DCG—Eq. 8) [60].

Another important issue in the evaluation of a RS is the splitting method used for dividing the dataset into training and testing set. The most used methods are hold-out and cross-validation. In the hold-out method, the dataset is divided into $\alpha \%$ for training and $1-\alpha \%$ for testing. In the cross-validation method, the dataset is divided into q equal sets, and in each evaluation, we use q − 1 sets as training data and 1 set as testing data. Each evaluation has different sets of the dataset, ensuring that all the dataset is tested, and avoiding over-fitting. This method does not require a validation set [61]. The validation set is only required when cross-validation is used simultaneously for selection of the best set of hyperparameters and for error estimation [62], which is not our case and of many other related works on recommender systems [7, 37, 41].

## Experiments

For this work, we used a preexisting dataset, called CheRM-20, which was created by [5, 63]. The CheRM-20 is a recommendation dataset with the standard format of <user,item,rating>. According to the authors, the dataset was developed using a methodology called LIBRETTI, which allows the creation of standard recommendation datasets by using research literature for extracting implicit feedback for the researchers. Thus, in CheRM-20, the users are authors from research papers, the items are chemical compounds, which may be linked to ChEBI ontology, and the ratings are the number of articles an author wrote about a chemical. With CheRM-20, we have access to information about the researchers’ past interests for chemical compounds, which allows us to develop recommender algorithms for predicting which chemical compounds the researchers may be interested now, based on their past ratings and the ratings of their similar peers.

CheRM-20 has 16.437 items, 2.193 users, and 117.020 ratings. All the users in the dataset have rated at least 20 items, i.e., the researchers considered in this dataset wrote articles about at least 20 of the 16.437 chemical compounds, even if only one article per item. This condition imposes a minimum number of items per user and it serves the sole purpose of when splitting the dataset into train and test, both datasets have a minimum number of items, providing a fair evaluation. This is a recurrent practice in other recommendation datasets, such as MovieLens [6]. On the contrary, there is no limitation for the minimum number of authors rating an item, which is an advantage because an item with only one rating (only one author wrote one paper about this chemical compound) has still the possibility of being recommended. Since this dataset’s rating was collected from implicit feedback, we will use algorithms suitable for this kind of data, such as ALS and BPR.

Table 1 shows the variation of algorithms evaluated in this study. For CF, we tested ALS and BPR, separately. We tested different latent factors, achieving the best results for this data with 150 factors. For CB, we tested the ONTO algorithm, using three different similarity measures: Lin, Resnik, and JC. The Hybrids were developed in combinations of the CF and CB approaches, using the two different metrics for calculating the final score of each item in the test set, Metric1—Eq. 2 and Metric2—Eq. 3.

**Table 1:** Variation of the algorithms evaluated

CF	CB	Metric	Algorithm
ALS	–	–	ALS
BPR	–	–	BPR
–	ONTO_JC	–	ONTO_JC
–	ONTO_LIN	–	ONTO_LIN
–	ONTO_RESNIK	–	ONTO_RESNIK
ALS	ONTO_JC	Metric1	ALS_ONTO_JC_m1
ALS	ONTO_JC	Metric2	ALS_ONTO_JC_m2
ALS	ONTO_LIN	Metric1	ALS_ONTO_LIN_m1
ALS	ONTO_LIN	Metric2	ALS_ONTO_LIN_m2
ALS	ONTO_RESNIK	Metric1	ALS_ONTO_RESNIK_m1
ALS	ONTO_RESNIK	Metric2	ALS_ONTO_RESNIK_m2
BPR	ONTO_JC	Metric1	BPR_ONTO_JC_m1
BPR	ONTO_JC	Metric2	BPR_ONTO_JC_m2
BPR	ONTO_LIN	Metric1	BPR_ONTO_LIN_m1
BPR	ONTO_LIN	Metric2	BPR_ONTO_LIN_m2
BPR	ONTO_RESNIK	Metric1	BPR_ONTO_RESNIK_m1
BPR	ONTO_RESNIK	Metric2	BPR_ONTO_RESNIK_m2

We used offline methods for evaluating the performance of the algorithms for the top@k, with k varying between 0 and 20, with steps of 1 [59]. From the vast range of metrics for evaluating recommender algorithms, we selected classification accuracy metrics and rank accuracy metrics, since they allow us to evaluate the algorithms for the relevant and irrelevant items recommended in a ranked list, and for the ability of an algorithm to recommend the items in the correct order. We use Precision, Recall (classification accuracy metrics), MRR, and nDCG (rank accuracy metrics) for this study. All the selected evaluation metrics range between 0 and 1, with values closest to 1 better. For the segmentation of the dataset into training and testing sets, we used a 5 cross-validation approach, by splitting users and items into fivefolds. In each iteration we draw 20% of the users and 20% of the items as test data, and 80% as train data. We did not use a validation set, since it is not required when using a cross-validation approach. This split and evaluation method is used in several recommender system studies [7, 37, 41].

All the positive ratings in the test set are considered relevant items for the user, i.e., an item with a rating of 5 is not more relevant than an item with a rating of 1. If an author wrote one paper about one chemical compound, we consider this chemical relevant for the author. We considered the unrated items as negative ratings, i.e., not relevant for the users. For the ONTO algorithm, we also assessed how using the n most similar items affects the results, with n varying from 1, 5, 10, 15, 20, 25, 30, and all of the items.

**Fig. 3:**
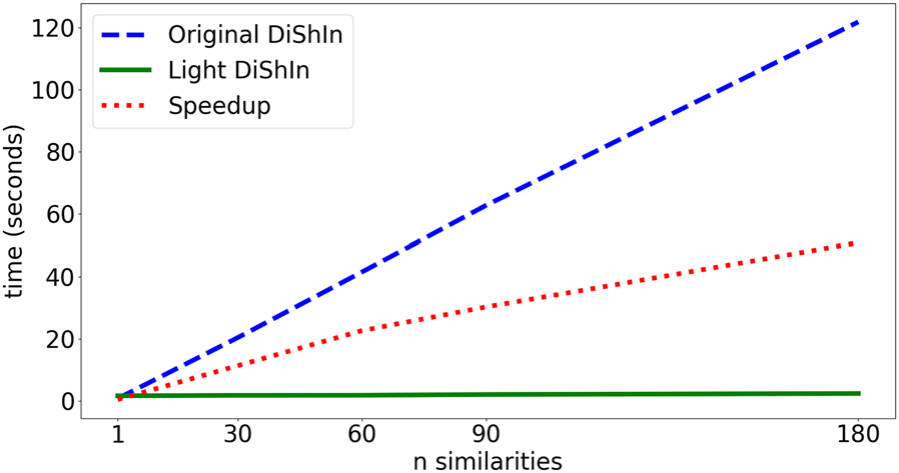
Light DiShIn speedup. Speedup of Light DiShIn with respect to the Original DiShIn

The semantic similarity between the chemical compounds was calculated offline, using the DiShIn. Despite DiShIn robustness, the framework was not fit for a large number of items. Thus, we implemented a new functionality, Light DiShIn, which allowed us to speedup the calculation of the similarities and the feasibility of the ONTO algorithm. Light DiShIn was implemented based on Pandas [64], which is a python Framework for manipulating datasets, and the use of multiprocessing, introducing the use of multiple cores for processing the similarities. Table 2 and Fig. 3 show the results of the speedup in latency (Eq. 10 [65]) of Light DiShIn when compared with the original DiShIn. The number of similarities calculated (n similarities) is 1, 30, 60 and 180, and both systems calculated Resnik, Lin, and JC similarity metrics.

10 $$Speedup_{Latency} = \frac{Latency1}{Latency2}$$

**Table 2:** Evaluation of the speedup latency from original DishIn to Light DiShIn

n similarities	Original DiShIn	Light DiShIn	Speed up
1	0.77	1.66	0.46
30	20.36	1.79	11.34
60	41.43	1.83	22.59
90	62.72	2.07	30.22
180	121.72	2.39	50.82

The latency is measured in seconds and n similarities is the number of similarities calculated in each iteration of the test

According to the results, for calculating the similarity between two entities (n similarities = 1), the original DiShIn is faster. Though, when increasing the number of entities and the number of similarities for calculation, the Light DiShIn is much faster than the original DiShIn, whose calculation time seems to be exponential. In our tests, the speedup latency from original DishIn to Light DiShIn achieves values of 50 times faster. For calculating the 131.538.810 similarities between the entities used for this work, we estimated that the original DiShIn would take 3.2 years. The similarities for 16.437 chemical compounds, 131.538.810 similarities, were calculated in less than a week and stored into a mySQL database for the measures Lin, Resnik and JC. This database is used by the ONTO algorithm for faster retrieving the semantic similarities of all items in the test and in the train sets. The introduction of Light DiShIn allows the viability of the execution of the ONTO algorithm, described in Algorithm 1.

## Results and discussion

**Fig. 4:**
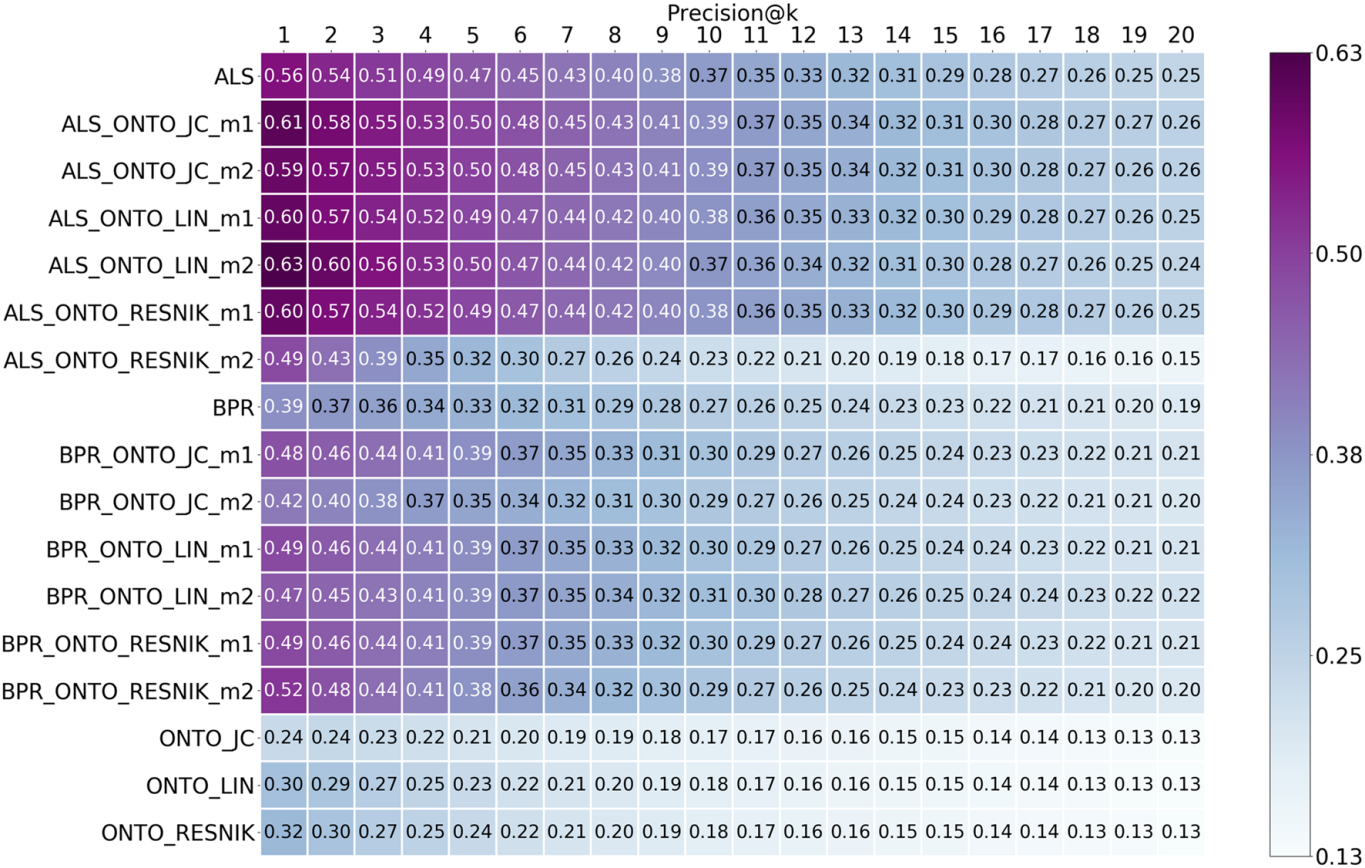
Precision results from top@1 to top@20, for ALS, BPR, ONTO and the Hybrids obtained using the 5 most similar items when calculating the scores for the ONTO algorithms

**Fig. 5:**
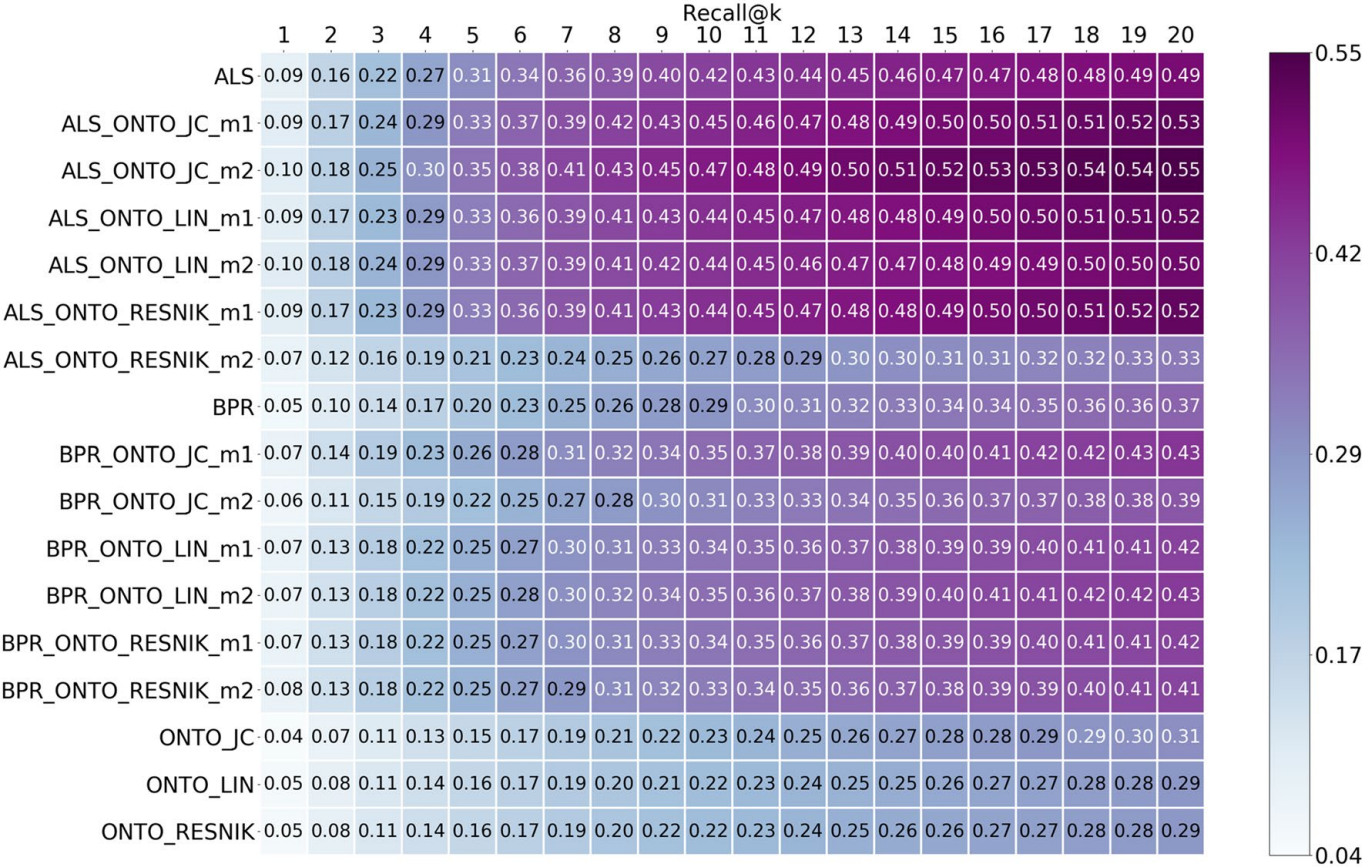
Recall results from top@1 to top@20, for ALS, BPR, ONTO and the Hybrids obtained using the 5 most similar items when calculating the scores for the ONTO algorithms

**Fig. 6:**
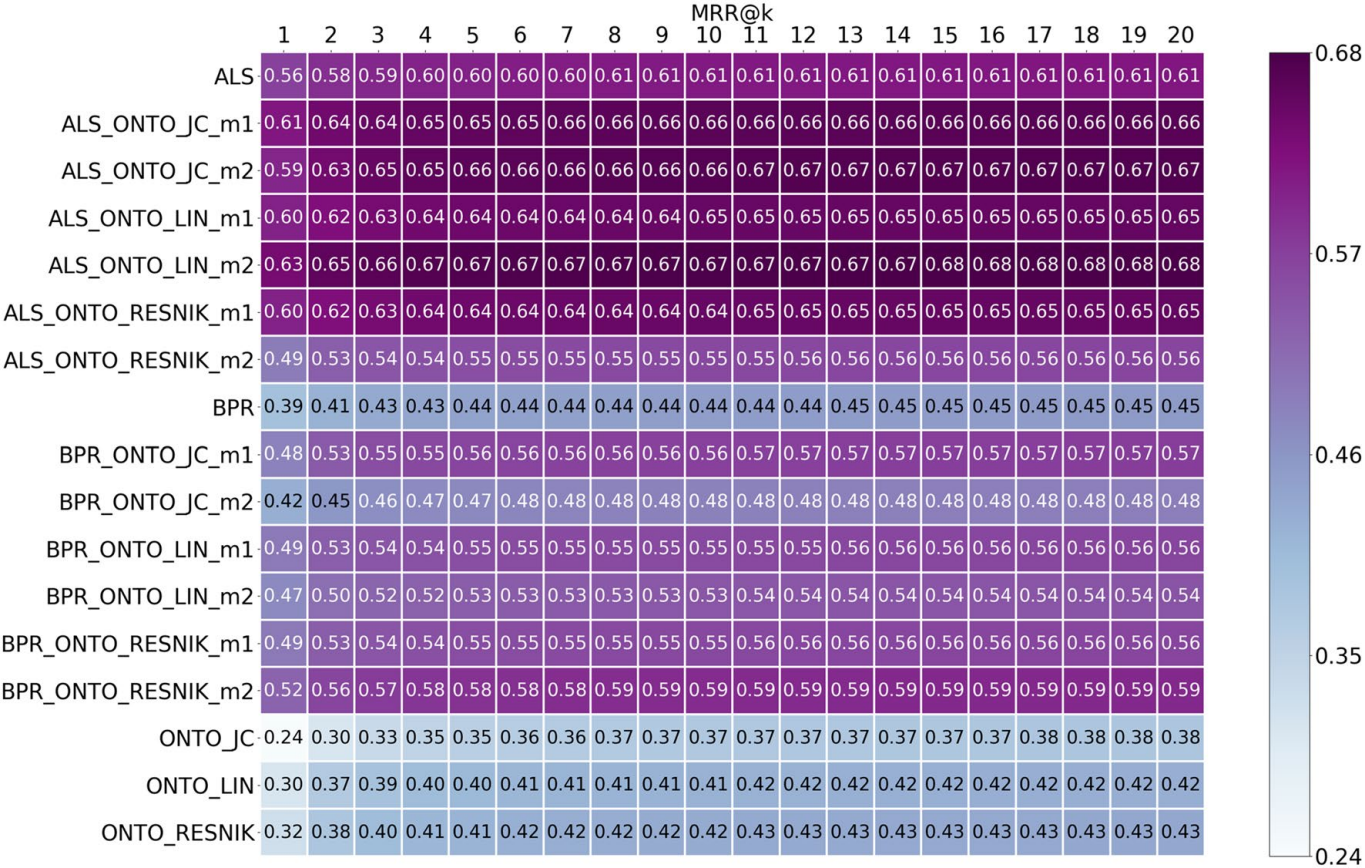
MRR results from top@1 to top@20, for ALS, BPR, ONTO and the Hybrids obtained using the 5 most similar items when calculating the scores for the ONTO algorithms

**Fig. 7:**
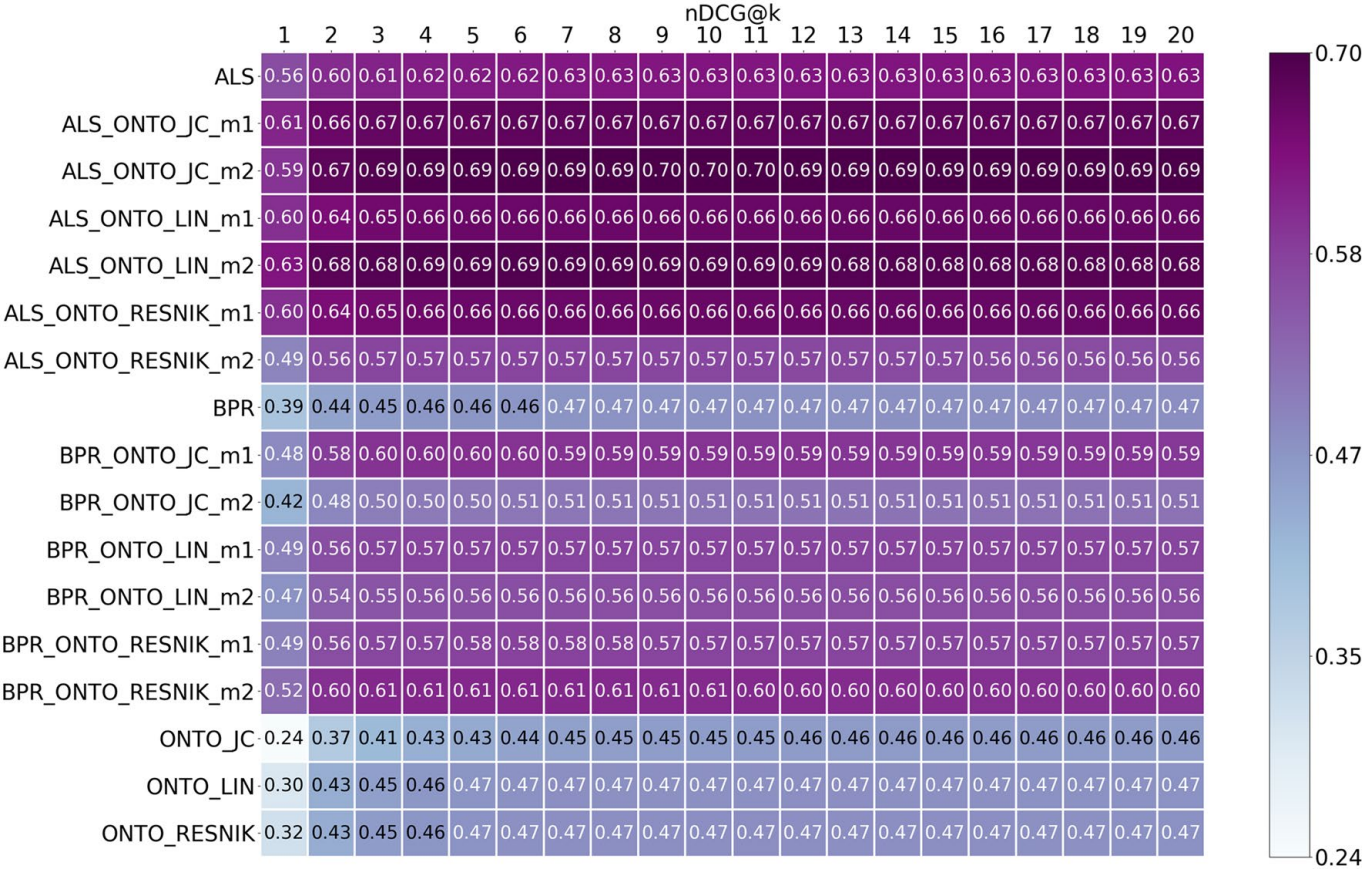
nDCG results from top@1 to top@20, for ALS, BPR, ONTO and the Hybrids obtained using the 5 most similar items when calculating the scores for the ONTO algorithms

We present the results of this study in Figs. 4, 5, 6, and 7 for Precision, Recall, MRR, and nDCG, respectively, through the form of heat-maps, for all the algorithms in Table 1. The heat-maps show the results from top@1 to top@20, obtained using the five most similar items when calculating the scores for the ONTO algorithm, since these were the best results obtained. Following the heat-map, the more purple, the better the results. The Hybrids, both with ALS and BPR, achieved the best values for all the represented metrics. The best precision was obtained with ALS-ONTO-LIN-m2 (0.63—top@1), improving ALS results by seven percentage points. The best recall was obtained with ALS-ONTO-JC-m2 (0.55—top@20), improving ALS results by six percentage points.

BPR had lower results than ALS for all the evaluated metrics. However, when combining BPR with ONTO, the improvement is more significant from BPR to BPR-ONTO than from ALS to ALS-ONTO. Precision had an improvement of 13 percentage points, and recall had an improvement of six percentage points. From these results, we may conclude that the combination of ALS with ONTO achieves the highest results, but the hybrids with BPR undergo more significant increases when compared to BPR alone. These results of precision and recall show that the Hybrid algorithms are including more relevant items in the list of recommendations.

Looking at the ranking quality metrics MRR and nDCG in Figs. 6 and 7, ALS-ONTO-LIN-m2 obtains the best MRR (0.68—top@15), with a growth of seven percentage points from ALS to ALS-ONTO-LIN-m2. ALS-ONTO-JC-m2 have the best nDCG (0.70—top@9,10,11), more seven percentage points than ALS. For BPR, the increase was 14 percentage points for MRR and 13 percentage points for nDCG. These results of MRR and nDCG indicate that the Hybrid algorithms are effective in rearranging the ranked list of recommendations.

Analysing Figs. 4, 5, 6 and 7, the ONTO algorithms alone have the lowest results in all evaluation metrics. Nevertheless, they follow the trend of the other algorithms, and when measuring these metrics for the top@20, the results are similar. ONTO has the advantage of being a CB algorithm; consequently, it does not have the problem of cold start for new items. ALS and BPR cannot be used if the item in the test set is not in the train set at least once (at least one author in the train set wrote about this chemical compound). However, ONTO algorithm requires the existence of all the entities in an ontology. In this case, the chemical compounds must be represented in ChEBI. When applying the ONTO algorithm to a database which does not have the ChEBI ID for the entities, we may use Named Entity Linking methods, such as the Relation Extraction for Entity Linking (REEL) [66], which links entities recognized in the literature to the ChEBI ontology.

ONTO-LIN and ONTO-RESNIK achieved almost the same results; however, the Hybrids created with the two metrics have quiet different results. The Hybrids with ALS created through Metric1 (Eq. 2) achieved similar results for both ONTO-LIN and ONTO-RESNIK. For Metric2, the Hybrids with ONTO-LIN are better (Eq. 3). The ranges of the scores may explain this. Whereas LIN has a range between 0 and 1, and ALS is also returning scores inferior to 1, the same is not true for ONTO-RESNIK, since the Resnik similarity metric has an infinite upper limit. Thus, when using Metric2 for calculating the final score for an item, the scores from ONTO-RESNIK have a much greater influence on the mean of the scores than the ones from ALS (<1).

For BPR, we verified that the Hybrid with ONTO-RESNIK with Metric1 achieved similar results to the ones obtained with ONTO-LIN. With Metric2, the Hybrid with ONTO-RESNIK is better than with ONTO-LIN. Due to BPR’s particularity, which always increments 1 to the scores, all scores for the items from this algorithm are higher than one. Between ALS and BPR, ALS achieved the best results. Since BPR is an algorithm for ranking, it was expected to obtain better results. We believe this is because the dataset has a large number of ratings equal to one, and many items have the same relevance (difficult to rank).

**Fig. 8:**
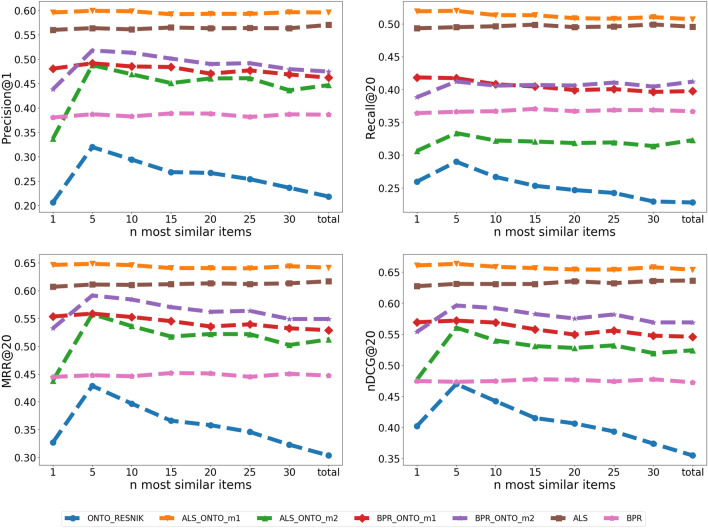
ONTO-RESNIK n variation. Variation of Precision@1, Recall@20, MRR@20 and nDCG@20 with different n most similar items in the ONTO-RESNIK algorithm

We will now see how the number n of most similar items is also influencing the results of the ONTO algorithm, as well as the results for the Hybrids. Figure 8 shows the variation in the Precision@1, Recall@20, MRR@20 and nDCG@20 with different n most similar items in the ONTO-RESNIK algorithm and for the Hybrids ALS-ONTO-RESNIK-m1, ALS-ONTO-RESNIK-m2, BPR-ONTO-RESNIK-m1, and BPR-ONTO-RESNIK-m2. ALS and BPR are also represented for better visualization of the improvement of the Hybrids. The small variations of ALS and BPR along the y axis are due to the stochastic nature of the evaluation methods.

Following Fig. 8, the best results for ONTO-RESNIK in all the evaluation metrics are achieved using the five most similar items for calculating the scores of the items in the test set. Using a higher n, the quality metrics decrease for all the evaluation metrics. These results also affect the Hybrid algorithms, lowering the quality metrics with the increase of n. ALS-ONTO-RESNIK-m1 is the best for all evaluation metrics. Looking at the plots in Fig. 8, we can notice a slightly descendent curve with the increase of the n most similar items. For example, the value for MRR@20 for ALS-ONTO-RESNIK-m2 is 0.6484 for n = 5 and 0.6460 for n = 10. This small difference may be because ALS has a much stronger influence on the final score than ONTO-RESNIK. As previously noticed, ALS-ONTO-RESNIK-m2 suffers a decrease when compared with ALS. This is justified by the different ranges of the scores for each algorithm, visibly affecting ALS-ONTO-RESNIK-m2 by the variation of n. BPR follows the trend of ALS results, with the difference that BPR-ONTO-RESNIK-m2 generally achieved best results than BPR-ONTO-RESNIK-m1.

**Fig. 9:**
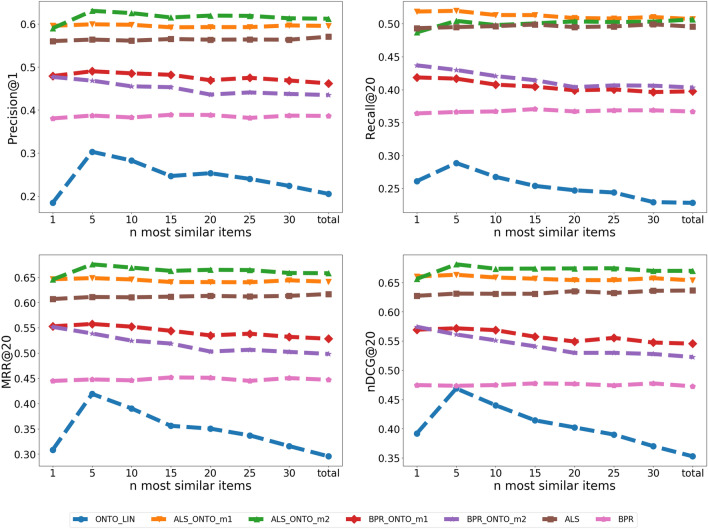
ONTO-LIN n variation. Variation of Precision@1, Recall@20, MRR@20 and nDCG@20 with different n most similar items in the ONTO-LIN algorithm

**Fig. 10:**
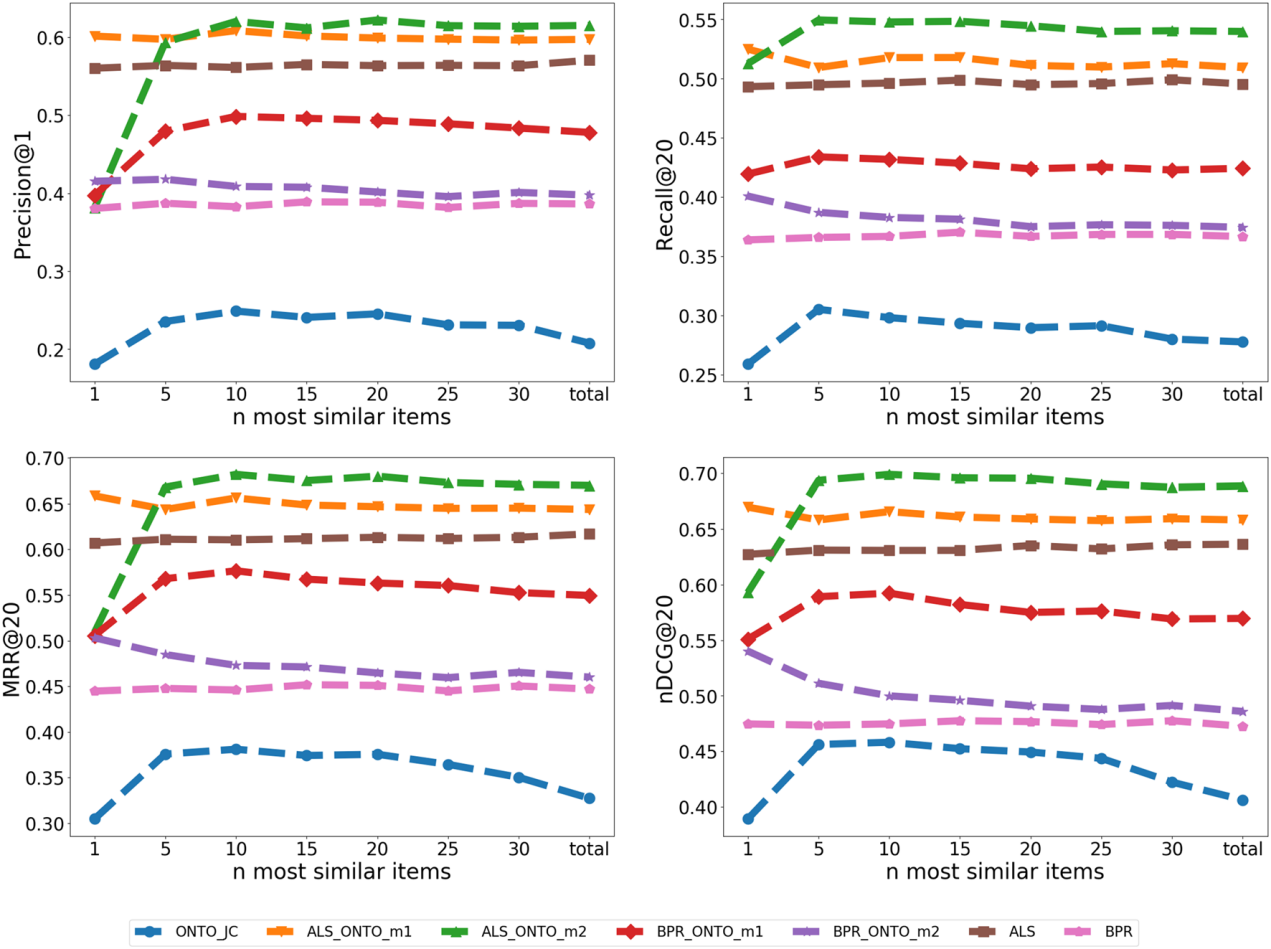
ONTO-JC n variation. Variation of Precision@1, Recall@20, MRR@20 and nDCG@20 with different n most similar items in the ONTO-JC algorithm

The results for the variation of the algorithms with the n most similar items for LIN and JC metrics are represented in Figs. 9 and 10, respectively. The analysis of the plots suggests the same behavior as the one for Resnik metric, i.e., the best results are achieved with n = 5, and they degrade with the increase of n.

The following example presented in Table 3 shows the influence of the ONTO-RESNIK algorithm in the order of the items in the ranked list of recommendations. The Table shows the top@20 recommended items with the algorithms ONTO-RESNIK, ALS, BPR, ALS-ONTO-RESNIK-m1 ALS-ONTO-RESNIK-m2, BPR-ONTO-RESNIK-m1 and BPR-ONTO-RESNIK-m2, for a user with ID 174228. This user has 4 relevant items in the test set, (ChEBI ID/name: 85291 (*N*,1,2-trioleoyl-sn-glycero-3- phosphoethanolamine (1-)), 85292 (*N*-stearoyl-1,2-dioleoyl-sn-glycero-3- phosphoethanolamine (1-)), 137008 (*N*-acyl-1-[(1Z)-alkenyl]-sn-glycero-3- phosphoethanolamine (1-)) and 140452 (1-[(1Z)-octadecenyl]-2-oleoyl-sn-glycero-3-phosphate (2)) i.e., items in the test set with a rating higher than zero. The relevant items recommended by each algorithm are represented in Italic cells. Additional info for all the chemical compounds mentioned in this text may be found in Additional file 1.

**Table 3:** Influence of the ONTO-RESNIK algorithm in the top@20 list of recommendations for user 174228

ONTO-RESNIK	ALS	BPR	ALS-ONTO-m1	ALS-ONTO-m2	BPR-ONTO-m1	BPR-ONTO-m2
85291	85292	23527	85292	85292	85292	85292
85292	85291	87818	85291	85291	85291	85291
85175	140452	72719	140452	85175	69120	140452
119	27847	6610	17697	119	140452	119
271436	175901	52347	5769	2904	137350	271436
2904	49668	72715	65495	271436	140243	6438
132187	87837	72754	27847	132187	132325	79079
79079	5769	69120	137411	79079	128770	132187
6438	17606	85292	49668	6438	69121	2904
140452	60453	140443	90983	140452	41214	69120
87764	87839	69340	132795	132725	82669	85175
132738	60747	132325	60999	132738	5635	137350
132725	76108	64499	30659	87764	63919	140243
65778	76097	140191	138802	78884	68249	128770
78884	60999	41214	138806	65778	69110	65778
76952	30659	91001	66917	141568	74912	69121
16108	31718	91000	37998	73275	140182	63919
77692	138802	133759	28850	138274	68236	69110
16125	138806	85291	66756	76952	130073	140182
31623	90983	67448	66755	16108	66394	130073

This user has as relevant items the following ChEBI IDs: 85291, 85292, 137008 and 140452. Underlined cells represent the relevant items recommended by each algorithm

**Fig. 11:**
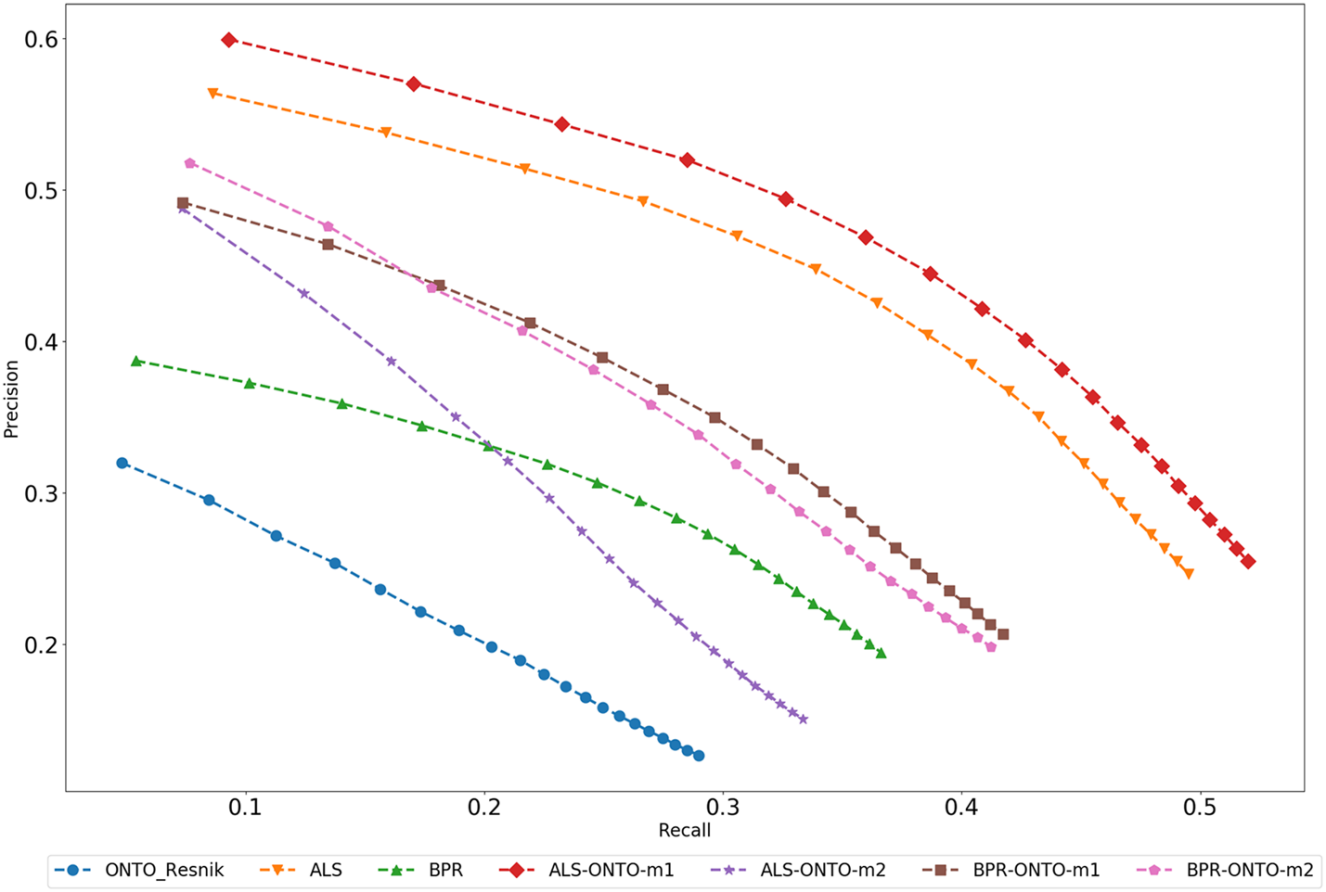
Precision-Recall curve. Precision-Recall curve for the algorithms ONTO-RESNIK, ALS, BPR, ALS-ONTO-m1, ALS-ONTO-m2, BPR-ONTO-m1, and BPR-ONTO-m2

For the example presented in Table 3, the best algorithms were ALS, ALS-ONTO-RESNIK-m1, and BPR-ONTO-RESNIK-m2, following the trend of our general results presented in Figs. 4, 5,  6, 7 and 8. Figure 11 shows the results for the Precision-Recall curve for all the algorithms in Table 1. This Figure shows that ALS-ONTO-m1 achieved the best results in the recommendation of the most relevant compounds.

When combining ONTO-RESNIK with ALS using the Metric1, the recommended items are the same, showing that for this case, ALS has a stronger influence in the final results. When combining ONTO-RESNIK with ALS using the Metric2, it results in the recommendation of less relevant items in the first positions of the list. The Hybrid of ONTO-RESNIK and BPR using Metric1 or Metric2 improves the number of relevant items recommended in the first positions for both BPR and ONTO-RESNIK. Based on these results, we may conclude that combining the ONTO algorithms with ALS or BPR, the most relevant items are rearranged for better positions in the Hybrids, improving the chances of recommending useful content for the users in the first positions of the recommendations. Thus, the results support our hypothesis that by using a CB algorithm based on the semantic similarity between the chemical compounds for creating Hybrids with CF algorithms, improves the recommendation of relevant items.

Considering that the size of the test set for this user was larger than 3000 items and the algorithms recommended three of the four relevant items in the first positions, one may say that RS are a solution for identifying chemical compounds of interest for scientific researches in large lists of these entities.

When using Model-based CF methods, it is not easy to justify why an item is recommended. However, our semantic approach finds a justification for the recommendations. Lets focus on Table 3, with the example for user 174228. The ChEBI IDs for the chemical compounds in the training set for this user were 134355, 137009, 137010, 137016, 137017, 138092, 138094, 138096, 140451, 61232, 62064, 62537, 71466, 78097, 78940, 85277, 85293, 85294, 85295, 85296, 85297, 85298, 85299, 85301, 85302, 85303, 85304, 85334 and 85335. The ONTO algorithm finds the semantic similarity between each item in the testing set (more than 3.000 items) and these items in the training set. The score for each item in the testing set is the mean of the similarity values. Thereby, for example, for item 85291, the score of ONTO-RESNIK is 4.67, being this the higher score for all 3.000 items in the test set. Interestingly, the score for item 85292 is also 4.67, which is justified by the fact that both items 85291 and 85292 are descendants of the item 62537, and share the same amount of common ancestors. This means that the items 85291 and 85292 share the most similarity with the items that we already know the user liked.

From a semantic and chemical point of view, both 85291 and 85292 are children of Organophosphate oxoanion (58945), which is an organic phosphoric acid, as well as a large number of compounds in the training set of this user—62537, 78097, 85277, 85293, 85294, 85295, 85296, 85297, 85298 and 85334. Thus, it makes sense that both are recommended to this user, and by the test set, these are true positives, because we know the user had interest in these compounds. Another large group of items in the training set of this user are Bronsted bases (molecular entity capable of accepting a hydron from a donor)—71466, 85299, 85301, 85302, 85303, 85304. The compound recommended by the ONTO algorithm in the third position (85175) is also a Bronsted base, thus, highly similar to these items in the training set. However, this compound is a false positive from the evaluation point of view, i.e., we don’t know if the user already had interest in this compound. Nevertheless, and based on the training set, if we recommend this item to the user, she/he will probably have interest in its study. This analysis is not possible for the CF algorithms. However, with the hybrids, we can also relate the items semantically and guide the user to study new compounds. For example, ALS-ONTO-m1 recommends in the fourth position the item 17697 (*N*-acetylserotonin). Despite this compound not being in the list of relevant items for this user, it is semantically similar to 85299 and 71466, which are from the group of Bronsted bases, and may be useful for this user research.

The only item in the list of relevant items which is not recommended by any algorithm is the 137008 (false negative). The reason this happens in the CF algorithms is because this item has a low number of users associated to it (3 users had interest in this item, the mean is 7 users by item). The ONTO algorithm is not able to retrieve this item in the list of recommendations due to a limitation of the DiShIn. The ID 137008 is a secondary ID for the compound 140403 (name:* N*-acyl-1-[(1Z)-alkenyl]-sn-glycero-3-phosphoethanolamine(1-)) and DiShIn is not able to calculate the similarity for the secondary IDs because it only works with primary IDs.

Table 4 presents another example of recommendation using the ONTO-RESNIK algorithm, for the user 33142. In this example, we show the relevant items recommended and the relevant items not recommended in the top@20 list.

**Table 4:** Results of ONTO-RESNIK for the user 33142

Training	Relevant	Score	Top@20	Score
60561	***59484***	2.18	134258	7.59
62642	77367	6.82	61755	7.59
62664	77380	6.74	84082	7.59
62996	***77629***	6.60	84078	7.59
62997	84078	7.59	59949	7.48
62998	84082	7.59	90930	7.29
77314	***134230***	4.33	66139	7.29
77374			60381	6.87
77378			77367	6.82
77381			90775	6.82
77382			77380	6.74
77384			62471	6.65
77385			61847	6.65
77598			87452	6.65
77613			87799	6.65
77625			61713	6.65
77626			61329	6.65
77627			61334	6.65
77628			62534	6.65
84081			67164	6.65
84084				

The table presents the training items for this user, the relevant items in the testing set, the scores of these items calculated using the ONTO-RESNIK algorithm and the top@20 recommendations, and respective scores. Underlined are the relevant items which were recommended (77367, 77380, 84078, 84082) and in Bolditalic the relevant items which were not recommended in the top@20 (59484, 77629, 134230)

The relevant items recommended (77367, 77380, 84078, 84082) have a high semantic similarity with the items in the training set of this user. All the four are structural derivatives of oligosaccharide and carbohydrate. In the list of relevant items not recommended, we also have an item with these characteristics (77629); however, the score of this item is lower than the score of the last recommended item in the top@20, and that why it is not recommended. The other two items (59484 and 134230) do not share high semantic similarity with the train, explaining why they are not recommended.

Considering the results, the hybrid semantic recommender system presented in this work is suitable for the recommendation of chemical compounds of interest for researchers dealing with large scale datasets. The use of a hybrid approach not only improved the results of the individual module, but also provides recommendations of chemical compounds based on the interests of similar peers (CF) and being able of justifying the recommendation (CB).

The model described in this paper may also be applied to other databases in which it is possible to measure the semantic similarity between the entities. Consider the DrugBank [67], a major database of drugs, largely used in the pharmaceutical field. DrugBank, similarly to ChEBI, has chemical compounds, such as Acetaminophen. It provides detailed information about the chemicals, about their identification, pharmacology, or interactions, for example. It is also created in a hierarchical format, having a Chemical Taxonomy, which provides information such as **Super Class**, **Class**, **Sub Class**, and **Direct Parent**. This structure allows the calculation of semantic similarity between the chemicals, as shown in [68]. The ONTO algorithm can then be applied using these similarity measures for providing the recommendation, and combine it with other recommender algorithms such as ALS or BPR.

## Conclusion

A major challenge in the identification of new chemical compounds is the increasing number of entities added to repositories. In this work, we presented a solution to this problem in the form of a recommender system. Our approach consists of a Hybrid recommender model for recommending ranked lists of chemical compounds. The Hybrid model has two modules, one using a CF approach and the other a CB approach. In the CF module, we used ALS or BPR, specific algorithms for implicit feedback datasets. The CB module consists of a new algorithm called ONTO, based on the semantic similarity of the chemical compounds in ChEBI ontology. The hypothesis presented was that by combining the scores obtained by each module, we would improve the results of both modules separately. The Hybrids between ALS and ONTO were the ones with the best results for all the evaluation metrics, improving the results by more than ten percentage points. The obtained results support our hypothesis since the results for the Hybrids algorithms are higher when compared with the individual algorithms. Even though ALS and BPR are better than the ONTO versions of the CB approach, when combined, the ONTO algorithm rearranges the positions of the items, recommending more relevant items in the first positions of the rank. Thus, with this work, we contributed with a recommender framework for chemical compounds, a new CB semantic recommender algorithm based on ontologies, a new Hybrid recommender algorithm for datasets of implicit feedback, a dataset with the semantic similarity between more than 16.000 chemical compounds, and also a faster method for calculating the similarities between large numbers of entities. We believe that this work is suitable for other fields of study, thereby, for future work, we intend to assess the ONTO algorithm, as well as the Hybrids, with entities from other ontologies, such as GO and DO. We would like to improve the results for precision and recall, for example by performing Named entity recognition in the articles from where the CheRM-20 dataset was created, to have more items related to each user. Other hypotheses are testing other similarity metrics, and using the relations between the compounds to provide the recommendations.

## Supplementary information

**Additional file 1.** Structure of all the chemical compounds mentioned inthe manuscript. This additional file contains the ChEBI ID, the principal name and the structure for all the chemical compounds mentioned in this manuscript. The file is available at: https://github.com/lasigeBioTM/ChemRecSys/blob/master/chemical_compounds_structure.pdf.

## Data Availability

The datasets supporting the conclusions of this article are available in the ChemRecSys GitHub repository, https://github.com/lasigeBioTM/ChemRecSys.
