# Supplementary material for: Hybrid semantic recommender system for chemical compounds in large-scale datasets
Source: J Cheminform. 2021 Feb 23;13:15. doi: 10.1186/s13321-021-00495-2 (PMC7903631; doi:10.1186/s13321-021-00495-2)
Supplement: Supplementary file 1 — Additional file 1. Structure of all the chemical compounds mentioned inthe manuscript. This additional file contains the ChEBI ID, the principal name and the structure for all the chemical compounds mentioned in this manuscript. The file is available at: https://github.com/lasigeBioTM/ChemRecSys/blob/master/chemical_compounds_structure.pdf. [file 13321_2021_495_MOESM1_ESM.pdf]

ChEBI ID	Name	Structure
27732	caffeine	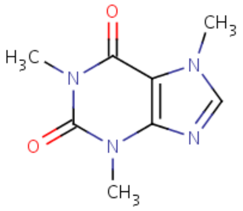
85291	N,1,2-trioleoyl-sn-glycero-3-phosphoethanolamine(1−)	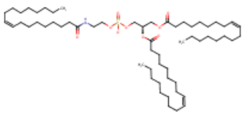
85292	N-stearoyl-1,2-dioleoyl-sn-glycero-3-phosphoethanolamine(1−)	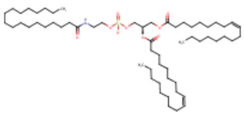
137008	N-acyl-1-[(1Z)-alkenyl]-sn-glycero-3-phosphoethanolamine(1−)	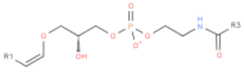
140452	1-[(1Z)-octadecenyl]-2-oleoyl-sn-glycero-3-phosphate(2−)	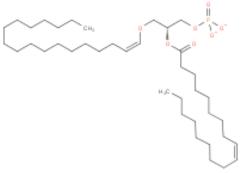
134355	auraptene	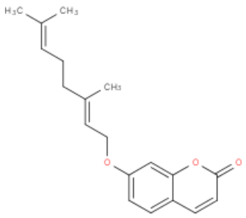
137009	1-(1Z-octadecenyl)-sn-glycero-3-phospho-(N-hexadecanoyl) ethanolamine(1−)	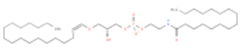

137010	1-(1Z-octadecenyl)-sn-glycero-3-phospho-(N-oleoyl)ethanolamine (1−)	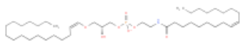
137016	1-(1Z-octadecenyl)-sn-glycero-3-phospho-(N-arachidonoyl) ethanolamine(1−)	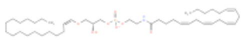
137017	1-(1Z-octadecenyl)-sn-glycero-3-phosphate(2−)	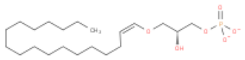
138092	1-[(Z)-alk-1-enyl]-sn-glycero-3-phospho-(N-acyl)ethanolamine	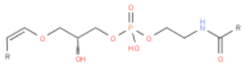
138094	1-(1Z-octadecenyl)-sn-glycero-3-phospho-(N-hexadecanoyl) ethanolamine	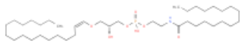
138096	1-(1Z-octadecenyl)-sn-glycero-3-phospho-(N-arachidonoyl) ethanolamine	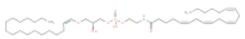
140451	N-acyl-1-[(1Z)-alkenyl]-2-acyl-sn-glycero-3-phosphoethanolamine(1−)	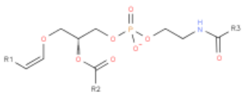
61232	N-acylphosphatidylethanolamine	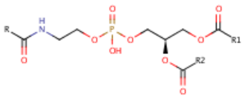

62064	butane-2,3-diol	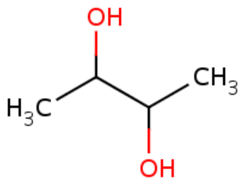
62537	N-acylphosphatidylethanolamine(1−)	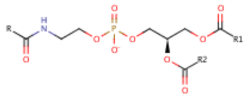
71466	oleoyl ethanolamide	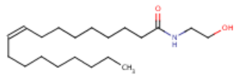
78097	N-palmitoyl-1,2-dioleoyl-sn-glycero-3-phosphoethanolamine(1−)	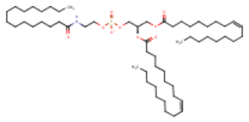
78940	N-palmitoyl-1,2-dioleoyl-sn-glycero-3-phosphoethanolamine	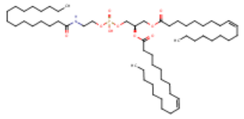
85277	N-arachidonoyl-1,2-dioleoyl-sn-glycero-3-phosphoethanolamine(1−)	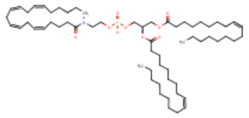
85293	N-myristoyl-1,2-dioleoyl-sn-glycero-3-phosphoethanolamine(1−)	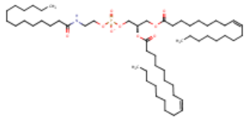
85294	N-lauroyl-1,2-dioleoyl-sn-glycero-3-phosphoethanolamine(1−)	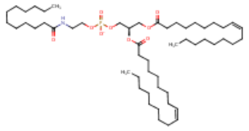

85295	N-capryl-1-palmitoyl-2-linoleoyl-sn-glycero-3-phosphoethanolamine(1−)	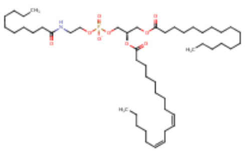
85296	N-capryloyl-1-palmitoyl-2-linoleoyl-sn-glycero-3-phosphoethanolamine (1−)	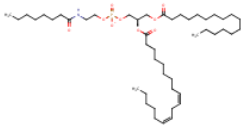
85297	N-caproyl-1-palmitoyl-2-linoleoyl-sn-glycero-3-phosphoethanolamine(1−)	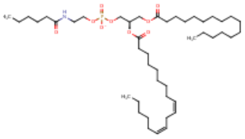
85298	N-butyryl-1-palmitoyl-2-linoleoyl-sn-glycero-3-phosphoethanolamine(1−)	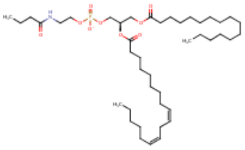
85299	N-(octadecanoyl)ethanolamine	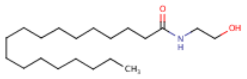
85301	N-(decanoyl)ethanolamine	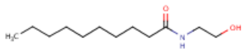
85302	N-(octanoyl)ethanolamine	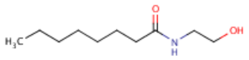
85303	N-(hexanoyl)ethanolamine	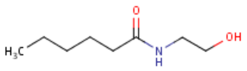

85304	N-(butanoyl)ethanolamine	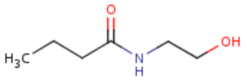
85334	N,1-dipalmitoyl-2-linoleoyl-sn-glycero-3-phosphoethanolamine(1−)	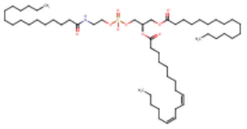
85335	N,1-dipalmitoyl-sn-glycero-3-phosphoethanolamine(1−)	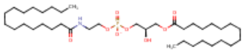
85175	11β-prostaglandin F2α ethanolamide	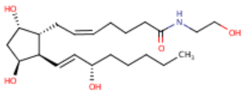
119	D-synephrine	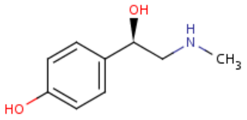
271436	N,N-dimethylethanolamine	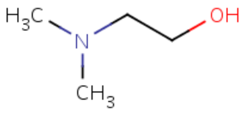
2904	atenolol	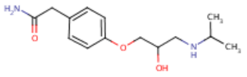
132187	N-ethyldiethanolamine	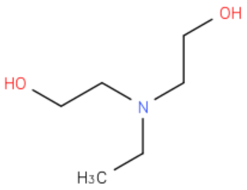

79079	(4S)-4-hydroxy-L-isoleucine	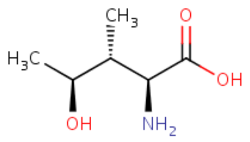
6438	levobunolol	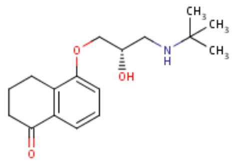
87764	N-docosanoylglycine	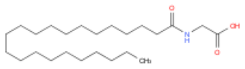
132738	N-[(15S)-hydroperoxy-(5Z,8Z,11Z,13E)-icosatetraenoyl]glycine	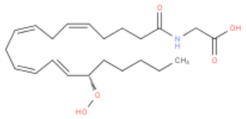
132725	N-[(12S)-hydroperoxy-(5Z,8Z,10E,14Z)-icosatetraenoyl]glycine	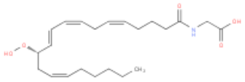
65778	16β,17-dihydroxy-ent-kaurane-19-oic acid	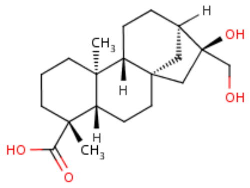
78884	4-terpineol	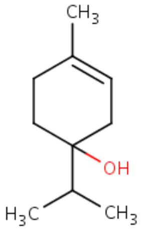
76952	syn-copalol	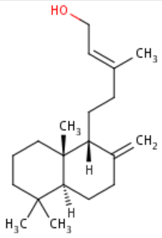

16108	dihydroxyacetone phosphate	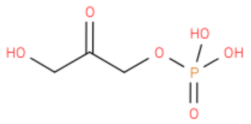
77692	peloruside A	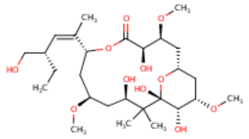
16125	hexadecan-1-ol	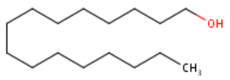
31623	fluocinolone acetonide	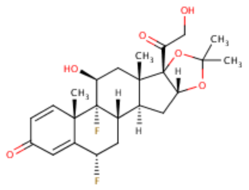
27847	3-iodo-L-tyrosine	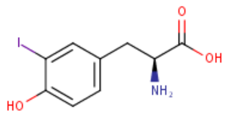
175901	gemcitabine	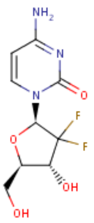
49668	gefitinib	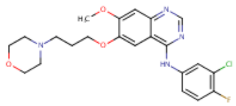
87837	ketocytochalasin	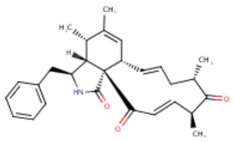

5769	humulone	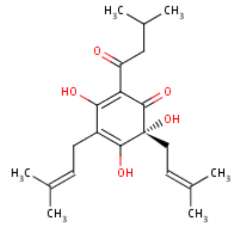
17606	diiodine	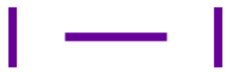
60453	2-O-oleoyl-3-O-palmitoyl-1-O-α-D-galactosyl-sn-glycerol	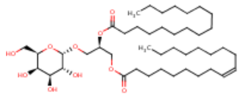
87839	cytochalasin Z16	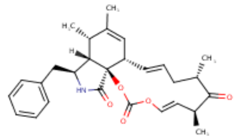
60747	α-Neu5Ac-(2→3)-β-Gal-(1→3)-β-GalNAc-(1→4)-β-Gal-(1→4)-β-Glc-(1↔1')-N-stearoylsphingosine	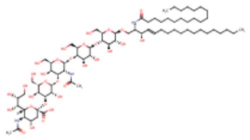
76108	1-O-[6-O-(1-naphthylcarbamoyl)-α-D-galactopyranosyl]-N-hexacosanoylphytosphingosine	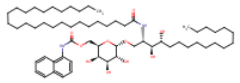
76097	1-O-{6-O-[(4-chlorophenyl)carbamoyl]-α-D-galactopyranosyl}-N-hexacosanoylphytosphingosine	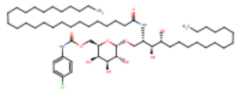
60999	3,5-diiodotyrosyl-3,5-diiodotyrosyl-3,5-diiodotyrosine	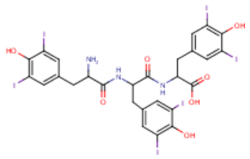

30659	D-thyroxine	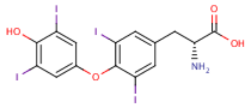
31718	ioxaglic acid	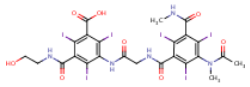
138802	Gly-Tyr-Ser-Ile-Thr-Ser-His-Tyr	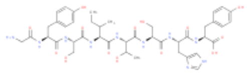
138806	Ala-Arg-Gly-Tyr-Ser-Ser-Phe-Arg-Tyr-Trp-Phe-Phe	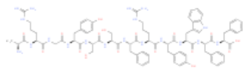
90983	(20S)-hydroxyvitamin D3	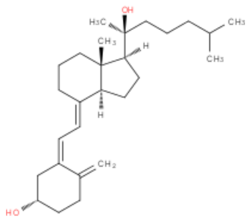
23527	cytochalasin B	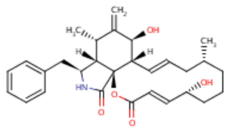
87818	sodium glycochenodeoxycholate	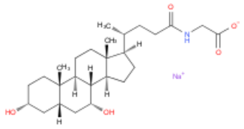
72719	4E,15Z-bilirubin IXa	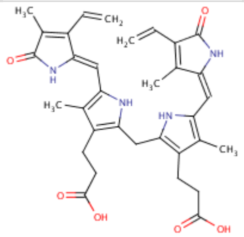

6610	lythramine	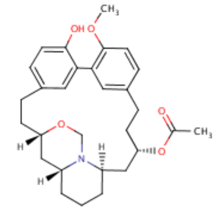
52347	3-hydroxyicosanoic acid	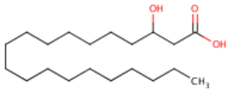
72715	O-linoelaidylcarnitine	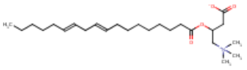
72754	bisphenol AF	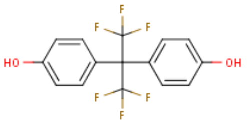
69120	withaferin A	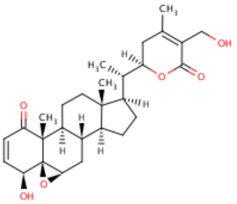
140443	viridicatin	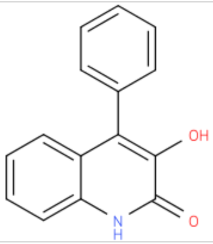
69340	ergolide	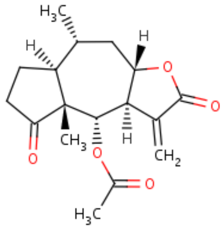
132325	21-HDoHE	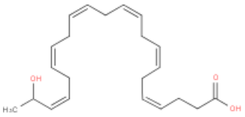

64499	shamixanthone	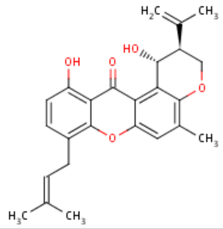
140191	17S,(16)-epoxy-(4Z,7Z,10Z,13Z, 19Z)-docosahexa-4,7,10,13,15,19-enoic acid	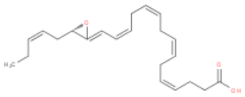
41214	4-nitrobenzyl alcohol	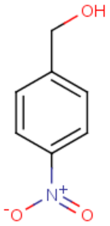
91001	18-oxoresolvin E1(1−)	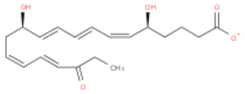
91000	resolvin E1(1−)	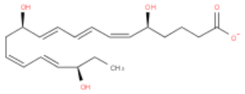
133759	fonsecinone A	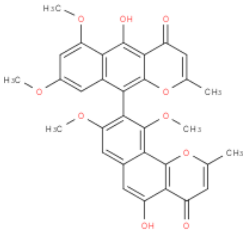
67448	ananolignan K	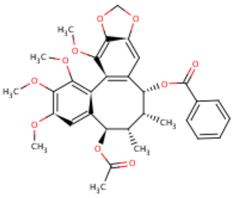
17697	N-acetylserotonin	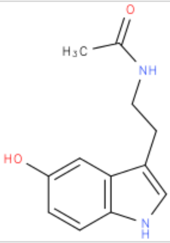

65495	bidenlignaside B	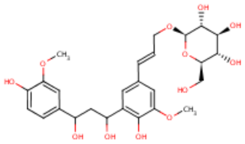
137411	1-hexadecanoyl-2-[(11Z,14Z,17Z)-icosatrienoyl]-sn-glycero-3-phosphocholine	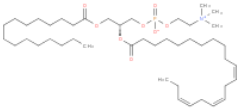
132795	lactinolide	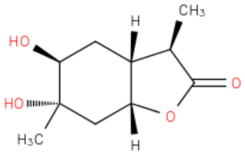
66917	ruxolitinib phosphate	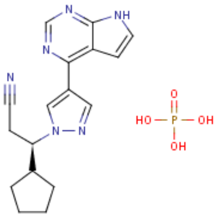
37998	glycolithocholic acid	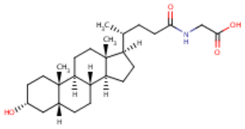
28850	dUDP	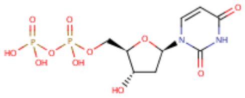
66756	phoyunbene D	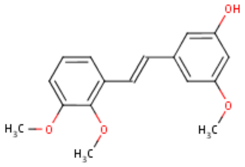
66755	phoyunbene C	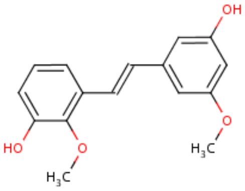

141568	N-ethyl-N-(2-hydroxyethyl) nitrosamine	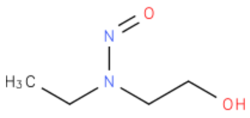
73275	ospemifene	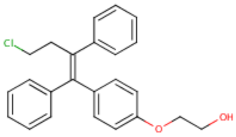
138274	14,15-epoxy-20-hydroxy-(5Z,8Z, 11Z)-icosatrienoic acid	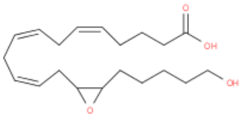
137350	(4Z,7Z,10Z,12E,14R,16Z,19Z) -14,22-dihydroxydocosahexaenoic acid	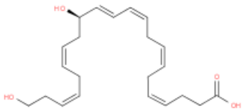
140243	22-hydroxyprotectin D1	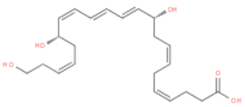
128770	12,18-dihydroxyoctadecanoic acid	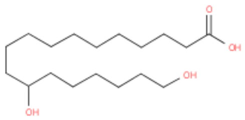
69121	2,3-dihydro-3β-methoxy withaferin A	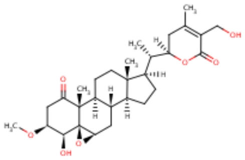
82669	AS-I-145	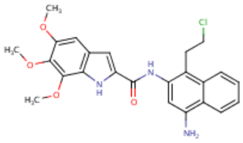

5635	helenalin	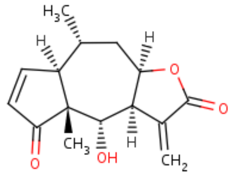
63919	3,3,4,4,5,5,6,6,7,7,8,8,9,9,10,10,10-heptadecafluoro-1-decanol	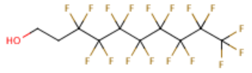
68249	platencin A1	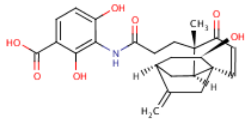
69110	withalongolide E	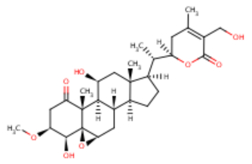
74912	N-{α-D-Man-(1→2)-α-D-Man-(1→2)-α-D-Man-(1→3)-[α-D-Man-(1→3)-[α-D-Man-(1→6)]-α-D-Man-(1→6)]-β-D-Man-(1→4)-β-D-GlcNAc-(1→4)-D-GlcNAc}-DHPE	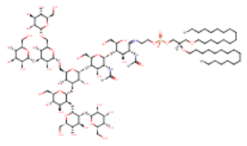
140182	hydroxyalbendazole	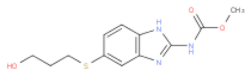
68236	platensimycin	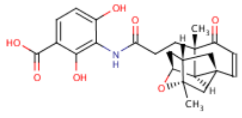
130073	5,20-diHEPE	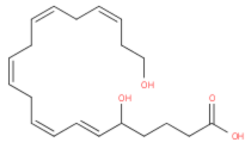

66394	minheryin G	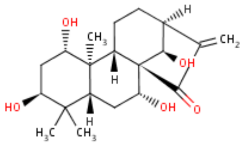
60561	D-Glcp-(1→3)-[α-D-GlcpNAc-(1→2)]-6-PEA-L-α-D-Hepp-(1→3)-L-α-D-Hepp-(1→5)-[α-Kdo-(2→4)]-α-Kdo	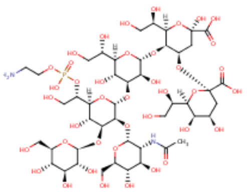
62642	β-D-Glc-(1→4)-[β-D-Glc-(1→3)]-[α-D-Glc-(1→2)-β-D-Glc-(1→6)]-α-D-Glc-(1→5)-[α-D-Kdo-(2→4)]-α-D-Kdo-(2→6)-β-D-GlcN4P-(1→6)-α-D-GlcN-1P	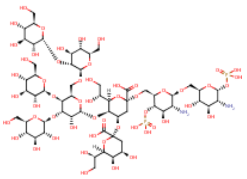
62664	α-D-GlcNAc-(1→2)-β-D-Glc-(1→4)-[β-D-Glc-(1→3)]-[α-D-Glc-(1→2)-β-D-Glc-(1→6)]-α-D-Glc-(1→5)-[α-D-Kdo-(2→4)]-α-D-Kdo-(2→6)-β-D-GlcN4P-(1→6)-α-D-GlcN-1P	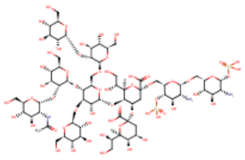
62996	α-D-GlcNAc-(1→2)-β-D-Glc-(1→4)-[β-D-Glc-(1→3)]-[α-D-Gal-(1→4)-β-D-Gal-(1→4)-α-D-Glc-(1→2)-β-D-Glc-(1→6)]-α-D-Glc-(1→5)-[α-D-Kdo-(2→4)]-α-D-Kdo-(2→6)-Lipid A	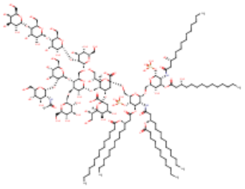
62997	α-D-Gal-(1→4)-β-D-Gal-(1→4)-α-D-Glc-(1→2)-β-D-Glc-(1→4)-[β-D-Glc-(1→3)]-[α-D-Gal-(1→4)-β-D-Gal-(1→4)-α-D-Glc-(1→2)-β-D-Glc-(1→6)]-α-D-Glc-(1→5)-[α-D-Kdo-(2→4)]-α-D-Kdo-(2→6)-Lipid A	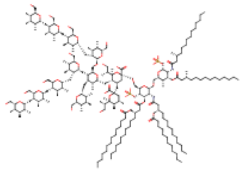
62998	α-D-Gal-(1→4)-β-D-Gal-(1→4)-α-D-GlcNAc-(1→2)-β-D-Glc-(1→4)-[β-D-Glc-(1→3)]-[α-D-Gal-(1→4)-β-D-Gal-(1→4)-α-D-Glc-(1→2)-β-D-Glc-(1→6)]-α-D-Glc-(1→5)-[α-D-Kdo-(2→4)]-α-D-Kdo-(2→6)-Lipid A	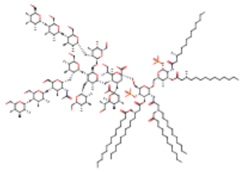
77314	β-D-Galp-(1→4)-β-D-GlcpNac-(1→3)-β-D-Galp-(1→4)-β-D-Glcp-(1→4)-[α-D-GlcpNac-(1→2)-L-α-D-Hepp-(1→3)]-L-α-D-Hepp-(1→5)-Kdo	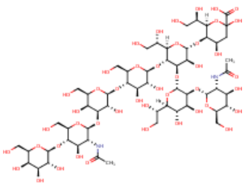

77374	β-D-GlcpNAc-(1→3)-β-D-Galp-(1→4)-β-D-Glcp-(1→4)-[α-D-GlcpNAc-(1→2)-3-PEA-L-α-D-Hepp-(1→3)]-L-α-D-Hepp-(1→5)-Kdo	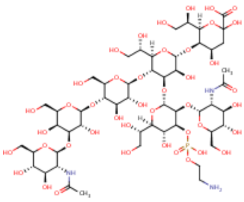
77378	β-D-GlcpNAc-(1→3)-β-D-Galp-(1→4)-β-D-Glcp-(1→4)-[α-D-GlcpNAc-(1→2)-L-α-D-Hepp-(1→3)]-L-α-D-Hepp-(1→5)-Kdo	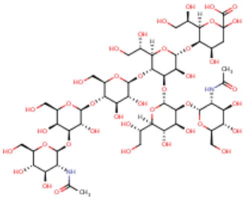
77381	β-D-Glcp-(1→4)-[α-D-GlcpNAc-(1→2)-3-PEA-L-α-D-Hepp-(1→3)]-L-α-D-Hepp-(1→5)-Kdo	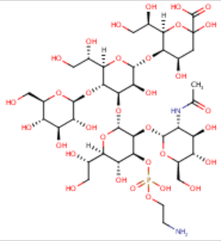
77382	β-D-Glcp-(1→4)-[α-D-GlcpNAc-(1→2)-L-α-D-Hepp-(1→3)]-L-α-D-Hepp-(1→5)-Kdo	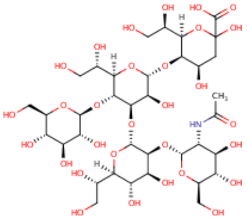
77384	α-D-GlcpNAc-(1→2)-3-PEA-L-α-D-Hepp-(1→3)-L-α-D-Hepp-(1→5)-Kdo	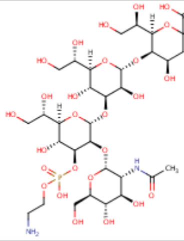
77385	α-D-GlcpNAc-(1→2)-L-α-D-Hepp-(1→3)-L-α-D-Hepp-(1→5)-Kdo	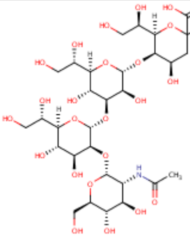
77598	α-D-GlcNAc-(1→2)-L-α-D-Hep-(1→3)-[β-D-Glc-(1→4)]-L-α-D-Hep-(1→5)-[α-D-Kdo-(2→4)]-α-D-Kdo	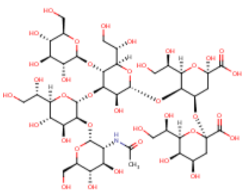
77613	α-D-GlcpNAc-(1→2)-3-PEA-L-α-D-Hepp-(1→3)-[β-D-Glcp-(1→4)]-L-α-D-Hepp	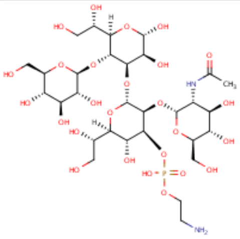

77625	α-D-GlcpNAc-(1→2)-L-α-D-Hepp-(1→3)-[β-D-Glcp-(1→4)]-L-α-D-Hepp	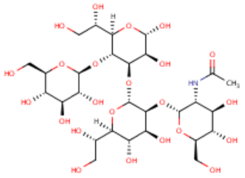
77626	α-D-GlcpNAc-(1→2)-[α-D-Glcp-(1→3)]-L-α-D-Hepp-(1→3)-[β-D-Glcp-(1→4)]-L-α-D-Hepp	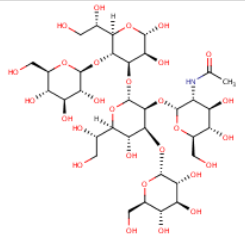
77627	α-D-GlcpNAc-(1→2)-6-PEA-L-α-D-Hepp-(1→3)-[β-D-Glcp-(1→4)]-L-α-D-Hepp	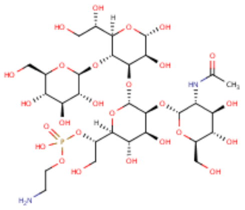
77628	α-D-GlcpNAc-(1→2)-[α-D-Glcp-(1→3)]-6-PEA-L-α-D-Hepp-(1→3)-[β-D-Glcp-(1→4)]-L-α-D-Hepp	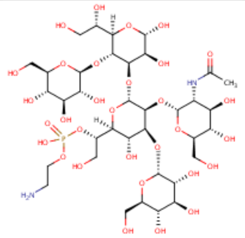
84081	β-D-Glcp-(1→4)-[α-D-GlcpNAc-(1→2)-3-PEA-L-α-D-Hepp-(1→3)]-L-α-D-Hepp-(1→5)-α-Kdo-(2→6)-(de-O-acylated lipid A)	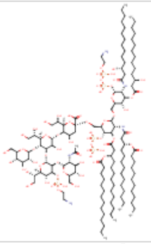
84084	3-PEA-L-α-D-Hepp-(1→3)-L-α-D-Hepp-(1→5)-α-Kdo-(2→6)-(de-O-acylated lipid A)	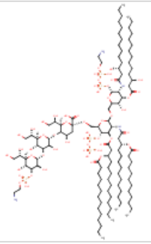
59484	α-D-Galp-(1→4)-β-D-Galp-(1→4)-β-D-Glcp-(1→4)-β-D-Glcp	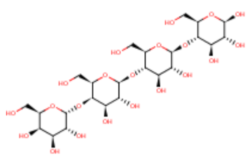
77367	β-D-Galp-(1→4)-β-D-GlcpNAc-(1→3)-β-D-Galp-(1→4)-β-D-Glcp-(1→4)-[α-D-GlcpNAc-(1→2)-3-PEA-L-α-D-Hepp-(1→3)]-L-α-D-Hepp-(1→5)-Kdo	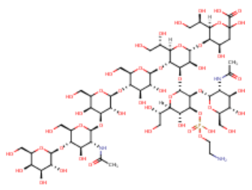

77380	β-D-Galp-(1→4)-β-D-Glcp-(1→4)-[α-D-GlcpNAc-(1→2)-3-PEA-L-α-D-Hepp-(1→3)]-L-α-D-Hepp-(1→5)-Kdo	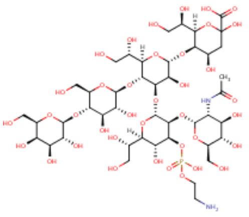
77629	α-D-GlcpNAc-(1→2)-3,6-diPEA-L-α-D-Hepp-(1→3)-[β-D-Glcp-(1→4)]-L-α-D-Hepp	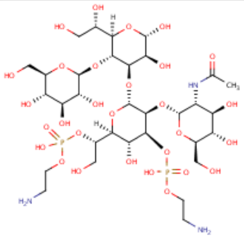
84078	β-D-Galp-(1→4)-β-D-GlcpNAc-(1→3)-β-D-Galp-(1→4)-β-D-Glcp-(1→4)-[α-D-GlcpNAc-(1→2)-3-PEA-L-α-D-Hepp-(1→3)]-L-α-D-Hepp-(1→5)-α-Kdo-(2→6)-(de-O-acylated lipid A)	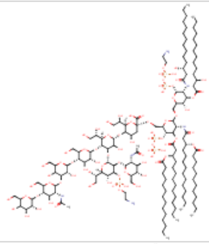
84082	α-D-GlcpNAc-(1→2)-3-PEA-L-α-D-Hepp-(1→3)-L-α-D-Hepp-(1→5)-α-Kdo-(2→6)-(de-O-acylated lipid A)	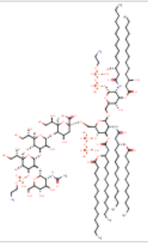
134230	α-L-FucpNAc-(1→3)-α-D-GalpNAc-(1→4)-α-D-GalpO[CH2]5NH2	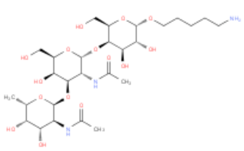
134258	β-L-Ara4N-lipid A (E. coli)	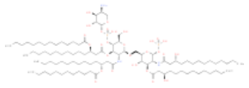
61755	β-L-Ara4N-(KDO)2-lipid IVA	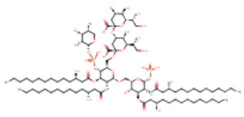
59949	N. meningiditis L7 lipoundecasaccharide	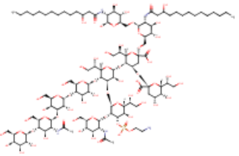

90930	aripiprazole lauroxil	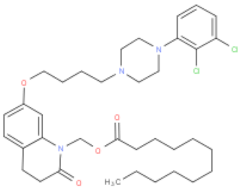
66139	kansuiphorin B	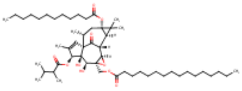
60381	α-D-Kdo-(2→4)-α-D-Kdo-(2→4)-α-D-Kdo-(2→6)-β-D-GlcNAc-4P-(1→6)-α-D-GlcNAc	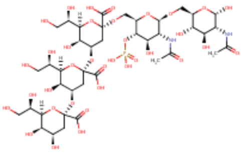
90775	α-D-Manp-(1→3)-[α-D-Manp-(1→6)]-α-D-Manp-(1→6)-[α-D-Manp6P-(1→2)-α-D-Manp-(1→3)]-β-D-Manp-(1→4)-β-D-GlcpNAc-(1→4)-D-GlcpNAc	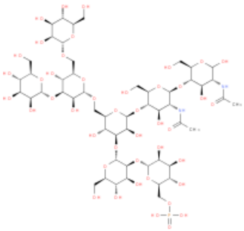
62471	α-L-Fucp-(1→2)-β-D-Galp-(1→4)-β-D-GlcpNAc-(1→3)-β-D-Galp-(1→4)-D-Glcp	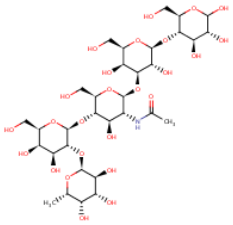
61847	β-D-Galp-(1→6)-β-D-Glcp-(1→6)-β-D-GlcpNAc-(1→3)-β-D-Galp-(1→4)-β-D-Glcp	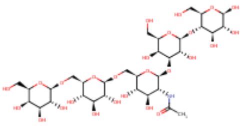
87452	β-D-Galp-(1→3)-β-D-GalpNAc-(1→3)-α-D-Galp-(1→4)-β-D-Galp-(1→4)-β-D-GlcpNAc	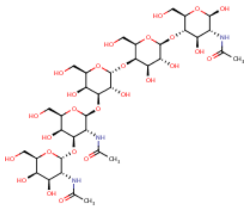
87799	α-L-Fucp-(1→2)-[α-D-Galp-(1→3)]-β-D-Galp-(1→4)-β-D-GlcpNAc-(1→6)-α-D-GalpNAc	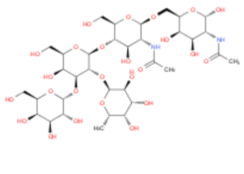

61713	α-Neup5Ac-(2→3)-β-D-Galp-(1→3)-β-D-GlcpNAc-(1→3)-[β-D-Glcp-(1→4)]-β-D-Galp	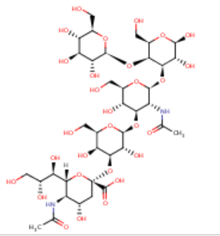
61329	α-L-Fucp-(1→2)-β-D-Galp-(1→4)-[α-L-Fucp-(1→3)]-β-D-GlcpNAc-(1→3)-β-D-Galp	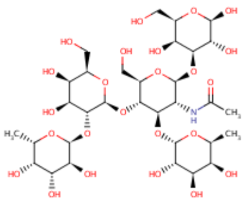
61334	α-L-Fucp-(1→4)-[β-D-Galp-(1→3)]-β-D-GlcpNAc-(1→3)-β-D-Galp-(1→4)-D-Glcp	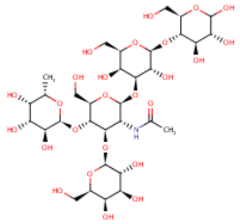
62534	α-L-Fucp-(1→2)-[α-D-GalpNAc-(1→3)]-β-D-Galp-(1→3)-β-D-GlcpNAc-(1→3)-β-D-Galp	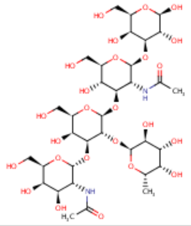
67164	α-L-Fucp-(1→2)-β-D-Galp-(1→4)-β-D-GlcpNAc-(1→3)-β-D-Galp-(1→4)-D-GlcpNAc	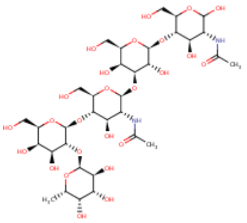
